# T47D Cells Expressing Myeloperoxidase Are Able to Process, Traffic and Store the Mature Protein in Lysosomes: Studies in T47D Cells Reveal a Role for Cys^319^ in MPO Biosynthesis that Precedes Its Known Role in *Inter*-Molecular Disulfide Bond Formation

**DOI:** 10.1371/journal.pone.0149391

**Published:** 2016-02-18

**Authors:** Richard P. Laura, David Dong, Wanda F. Reynolds, Richard A. Maki

**Affiliations:** 1 Torrey Pines Pharmaceuticals, Del Mar, California, United States of America; 2 Sanford-Burnham-Prebys Medical Discovery Institute, La Jolla, California, United States of America; Instituto de Biociencias - Universidade de São Paulo, BRAZIL

## Abstract

Among the human heme-peroxidase family, myeloperoxidase (MPO) has a unique disulfide-linked oligomeric structure resulting from multi-step processing of the pro-protein monomer (proMPO) after it exits the endoplasmic reticulum (ER). Related family members undergo some, but not all, of the processing steps involved with formation of mature MPO. Lactoperoxidase has its pro-domain proteolytically removed and is a monomer in its mature form. Eosinophil peroxidase undergoes proteolytic removal of its pro-domain followed by proteolytic separation into heavy and light chains and is a heterodimer. However, only MPO undergoes both these proteolytic modifications and then is further oligomerized into a heterotetramer by a single *inter*-molecular disulfide bond. The details of how and where the post-ER processing steps of MPO occur are incompletely understood. We report here that T47D breast cancer cells stably transfected with an MPO expression plasmid are able to efficiently replicate all of the processing steps that lead to formation of the mature MPO heterotetramer. MPO also traffics to the lysosome granules of T47D cells where it accumulates, allowing in-depth immunofluorescent microscopy studies of MPO trafficking and storage for the first time. Using this novel cell model we show that formation of MPO’s single *inter*-molecular disulfide bond can occur normally in the absence of the proteolytic events that lead to separation of the MPO heavy and light chains. We further demonstrate that Cys^319^, which forms MPO’s unique *inter*-molecular disulfide bond, is important for events that precede this step. Mutation of this residue alters the glycosylation and catalytic activity of MPO and blocks its entry into the endocytic pathway where proteolytic processing and disulfide bonding occur. Finally, using the endocytic trafficking of lysosomal hydrolases as a guide, we investigate the role of candidate receptors in the endocytic trafficking of MPO.

## Introduction

The enzyme myeloperoxidase (MPO) catalyzes the formation of hypochlorous acid (HOCl) from the substrates hydrogen peroxide (H_2_O_2_) and chloride and is one of several offensive weapons deployed by activated neutrophils. MPO is stored in an inactive state in the azurophil granules of neutrophils, a modified type of lysosome, and becomes catalytically productive only when a neutrophil is stimulated and turns on the enzyme nicotinamide adenine dinucleotide phosphate-oxidase [1, 2]. Nicotinamide adenine dinucleotide phosphate-oxidase provides high local concentrations of the otherwise limiting substrate H_2_O_2_ for the synthesis of HOCl by MPO. The chemical HOCl is a potent reactive oxygen species. The large number of circulating neutrophils combined with a high content of MPO creates the potential for significant reactive oxygen species-mediated damage to invading organisms but also to host proteins, lipids and DNA during an inflammatory response. Under conditions of chronic inflammation, especially in older individuals where cell repair pathways begin to fail, accumulated oxidative damage may lead to cell dysfunction and disease [3].

**Fig 1:**
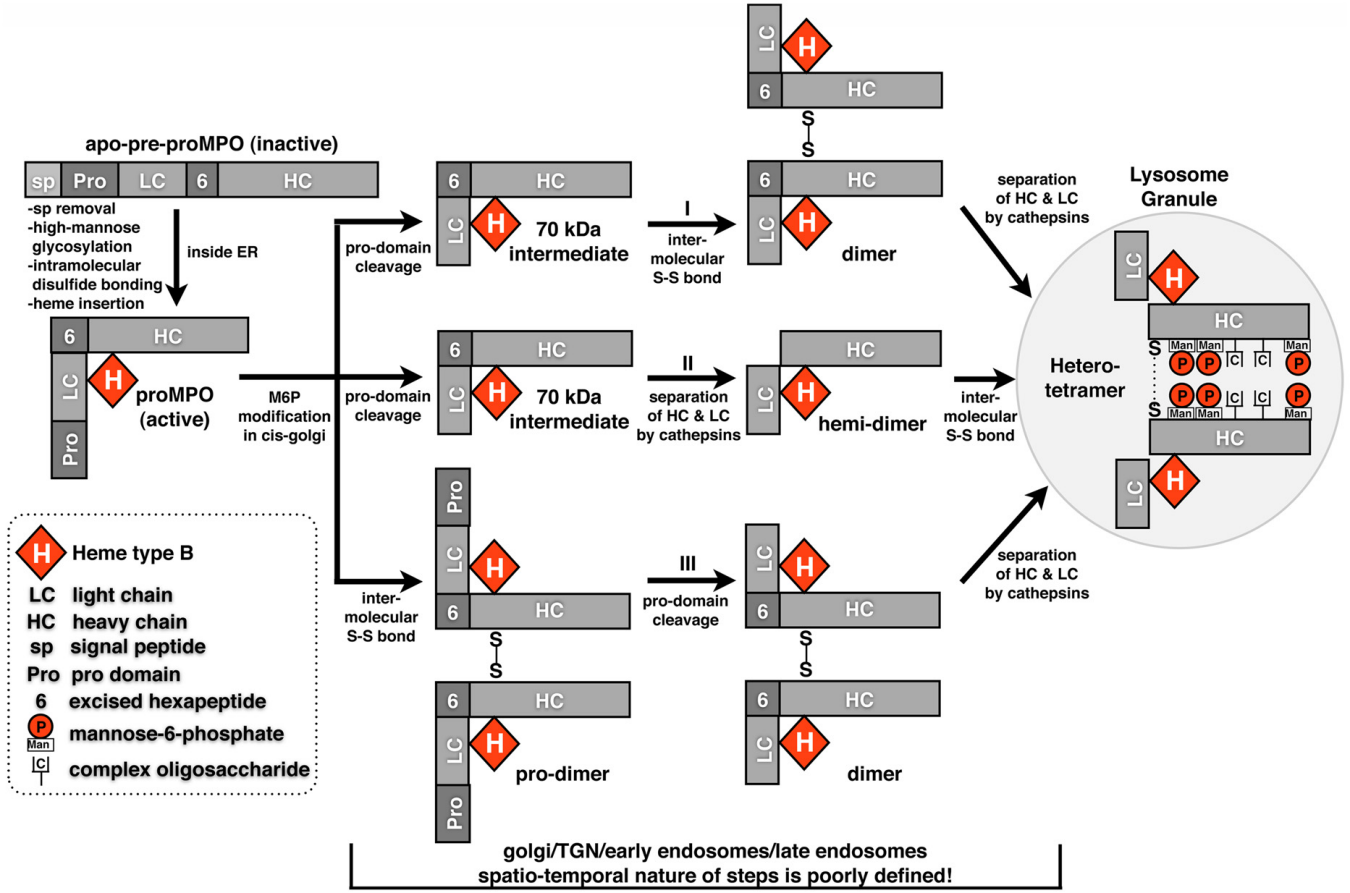
Outline of the steps in MPO processing based on our current knowledge. The general steps of MPO processing are known yet many details remain obscure. After MPO exits the endoplasmic reticulum (ER) both the order and precise subcellular localization of subsequent steps are unknown. Three possible scenarios are presented for the order of post-ER processing steps.

MPO is not transcribed or translated in neutrophils, but, along with other components of the azurophil granule is produced, processed and stored in promyelocytes within the bone marrow and prior to differentiation of these cells into neutrophils. MPO stored in neutrophils has a complicated heterotetrameric structure consisting of two heavy chains, two light chains and two heme prosthetic groups held together by distinct types of covalent bonds to form two symmetric catalytic units [4–6]. Each heavy-light chain pair is indirectly linked together into a dimer through covalent bonds with the shared heme prosthetic group. The two heavy-light chain pairs are, in turn, covalently linked by a single *inter-*molecular disulfide bond between the two heavy chains to form a tetramer. At a gross level one may see similarities to the domain structure of IgG, a major difference being that the heavy and light chains of MPO are not derived from separate gene products as are those of IgG, but are generated from a single gene product via proteolytic processing of a pro-protein monomer (proMPO) after its exit from the endoplasmic reticulum (ER). ProMPO is separated into heavy and light chains by at least three protease cleavage events (not including removal of the signal peptide) in which members of the furin and cathepsin protease families have been implicated, however, identification of the specific family members has yet to be accomplished (Fig 1). The *inter-*molecular disulfide bond of MPO is unusual in that it is not formed within the ER as are the *inter-*molecular disulfide bonds of IgG or as are the *intra-*molecular disulfide bonds that are present in the proMPO monomer but, occurs in an undefined organelle during transit to the lysosome granule (Fig 1). The spatio-temporal linkage between the protease cleavage events and formation of the *inter-*molecular disulfide bond has yet to be fully characterized.

Because of the abundance of the MPO heterotetramer stored in neutrophils, studying its enzymatic properties has been tractable, whereas, studying the multi-step processing and transport events that lead to formation of the heterotetramer in promyelocytes has been more complicated. The HL60 promyelocytic leukemia cell line replicates many of the attributes of promyelocytes and careful biochemical experimentation in these cells has produced much of what we currently understand about the biosynthesis of MPO. However, HL60 cells are constrained in the experimental approaches that can be applied to them, limiting their utility for further insights into the molecular mechanisms underlying MPO processing. Several recombinant expression systems for MPO have also been reported and have proven useful for characterization of the effects of disease-related MPO mutations on enzyme activity and some processing events, but, because of the limited amounts of heterotetramer produced by these expression systems, none of these cell models provides a practical option for examining the remaining questions surrounding biosynthesis of the MPO heterotetramer [4]. What has been missing is an experimentally tractable expression system that more efficiently recreates the complex processing, trafficking and storage events for MPO that occur in promyelocytes.

We report here two related breast cancer cell lines, T47D and MCF7, that when stably transfected with the MPO gene, generate the heterotetramer and store it in lysosomes with an efficiency approaching that of MPO-processing and storage in HL60 cells. Several other cell lines examined, including other breast cancer cell lines, produced little or no detectable heterotetramer. We validated the T47D cell model in detail by demonstrating the similarity of previously characterized traits of endogenously expressed MPO to those of recombinant MPO produced in these cells. We then show the versatility of this expression system by building on previous biochemical observations to gain new insights into the processing and trafficking of MPO. Specifically, using cathepsin inhibitors in the T47D-MPO expression system we show that separation of the heavy and light chains is not necessary for *inter-*molecular disulfide bond formation or for lysosomal storage of MPO. We then further probed the C^319^-C^319^ *inter-*molecular disulfide bond through mutagenesis to better define its co-dependence with other processing and trafficking events necessary for the formation and storage of the MPO heterotetramer. We show that mutation of C^319^ halts the proteolytic processing of MPO and interferes with trafficking, resulting in its accumulation within the Golgi. Additional observations of decreased peroxidase activity suggest that C^319^ may have a role in MPO processing that precedes its involvement in disulfide bonding. Finally, we carried out shRNA knockdown of known endocytic trafficking receptors in T47D-MPO cells to examine their role in the processing and trafficking of MPO. We firmly rule out any involvement of either cation-independent mannose-6 phosphate receptor (ciMPR) or cation-dependent mannose-6 phosphate receptor (cdMPR) consistent with previous studies. We also applied correlative mRNA expression analysis to recombinant and endogenous cell lines that can process and store MPO to determine whether any of the five VPS10p endocytic receptor family members was ubiquitously expressed. Only one member of this family, sortilin-related receptor (SorLA), was significantly expressed in all processing cell lines. Subsequent shRNA knockdown of SorLA in the T47D-MPO cells had no effect on MPO processing or trafficking and when combined with our quantitative PCR analysis, suggests that no member of the VPS10p family is likely to be involved in the biosynthetic processing and trafficking of MPO.

## Materials and Methods

### Reagents

NuPage 4–12% Bis-Tris SDS-PAGE gels and MOPS-SDS running buffer were from Life Technologies. Protran^™^ nitrocellulose (10600007) was from GE-Healthcare. Immobilon-P^™^ PVDF (IPVH00010) was from EMD-Millipore. Non-fat dry milk (B5001) was from Rockland, Inc. The enzymes PNGase F (P0704S), Endoglycosidase H (P0702S) and calf intestinal phosphatase (M0290S) were from New England Biolabs. The cathepsin inhibitors e64d (E8640) and N-acetyl-Leucine-Leucine-Norleucine (208719) were obtained from Sigma and Calbiochem respectively. Bovine liver catalase (C1345) was from Sigma. Complete^™^ protease inhibitor cocktail tablets with and without EDTA were from Roche (04693124001, 04693159001). The serine protease inhibitor PMSF (P7626) was from Sigma. Sureblue^™^ TMB microwell peroxidase reagent (52-00-03), used in the MPO ELISA and MPO activity assays, was from KPL, Kirkegaard & Perry Laboratories. The surfactants Tween-20 (P7949) and Triton X-100 (T8787) were from Sigma. The ECL reagent, WesternBright^™^ Sirius Western Blotting HRP substrate (K12043), was from Advansta and was used diluted 1 to 4 with water. Blue Devil^™^ autoradiography film (30–101) from Genesee Scientific was used for chemiluminescent development of immunoblots. Restore^™^ western blot stripping buffer (46430) from Thermo Scientific was used to strip blots prior to reprobing with a second antibody. Vectashield^™^ mounting media (H-1000) from Vector Labs was used to mount coverslips on slides for imaging. DAPI (4’, 6-diamidino-2-phenylindole) (D-1306) for fluorescent staining of nuclei was from Life Technologies. Lipofectamine 2000^™^ transfection reagent (11668) was obtained from Life Technologies. SP-Sepharose^™^ strong cation exchange resin (17-0729-10) was from GE Healthcare. IgG-depleted bovine serum albumin (A1311) was from US Biological. Normal donkey or horse serum (017-000-121, 008-000-121) were obtained from Jackson Immunoresearch Labs. Tissue culture media, additives and fetal bovine serum were from Corning, Inc. Geneticin^™^ (G418 sulfate, 61-234-RG) was obtained from Corning. Puromycin (P8833) was obtained from Sigma.

### Antibodies

The MPO antibodies used include a goat polyclonal from R&D Systems (AF3174) (immunofluorescence), rabbit polyclonal from DAKO (A0398) (ELISA-capture, MPO activity assay), and Mab-16E3 from Hytest (4M43) (immunoprecipitation and immunofluorescence). Horseradish peroxidase (HRP)-conjugate was generated from the goat polyclonal (immunoblot and ELISA detection) and Mab-16E3 (ELISA detection) anti-MPO antibodies using the Lightning-Link^™^ HRP-conjugation kit (701–0000) from Innova Biosciences. We also generated our own anti-MPO rabbit polyclonal antibody using purified neutrophil MPO (426–10) from Lee Biosciences as the antigen. Our rabbit polyclonal was used interchangeably with the goat polyclonal from R&D systems for immunoblot and immunofluorescence with no discernible difference in the results. Our rabbit polyclonal antibodies were also used as the source of heavy and light chain-specific antibodies. To isolate these, we ran 150 μg of purified neutrophil MPO on a single-well Tris-glycine SDS-PAGE gel under reducing conditions, transferred to PVDF and excised the PVDF strips containing each of the separated heavy and light chains. After blocking the strips with 4% non-fat dry milk in phosphate buffered saline (PBS), 0.05% Tween-20 and washing with PBS, 250 mM NaCl, 0.5% Triton X-100, the strips were incubated overnight at 4°C with immune serum diluted 1:10 in the wash buffer. The strips were washed extensively and the bound heavy or light chain-specific antibodies were eluted with 3 ml sodium citrate, pH 3.0. After neutralization with 300 μl of 1M Tris-base each eluate was dialyzed overnight at 4°C against PBS, centrifuged at 13,000 rpm to remove any precipitate and stored in aliquots at -80°C. The rabbit polyclonal anti-peptide antibody specific for the pro-domain of MPO was a gift from William Nauseef, M.D. (University of Iowa) and was generated against a peptide derived from sequence within the pro-domain of MPO. Organelle marker antibodies used in immunofluorescent cell staining against Lamp1 (D2D11), EEA1 (C45B10), RCAS1 (D2B6N) and PDI (C81H6) are rabbit monoclonal antibodies from Cell Signaling Technologies. The Mab to GM130 (610822) is from BD-Biosciences. Mab-10C3 to the KDEL sorting sequence (ADI-SPA-827) that is specific to resident proteins of the endoplasmic reticulum is from Enzo Life Sciences. Mab-22d4 against cdMPR and Mab-86f7 against ciMPR were obtained through the developmental studies hybridoma bank (DSHB) at the University of Iowa. Mab-22d4 recognizes both human and bovine cdMPR and Mab-86f7 recognizes only bovine but not human ciMPR [7]. Both MPR antibodies bind only the non-reduced forms of the respective receptors. The Mabs against E-cadherin (C20820) and SorLA (611860) are from BD-Transduction Labs. Mab-9E10 specific for the myc-epitope tag (M4439) was obtained from Sigma. The single chain variable fragment (ScFv) specific for mannose-6-phosphate was purchased from the Forschungsztrum Borstel Research Institute in Germany [8]. Secondary antibodies used for immunoblot were HRP-conjugated donkey anti-rabbit (NA934V) or sheep anti-mouse (NA931V) from GE Healthcare. Alexa-dye conjugated secondary antibodies used for immunofluorescent cell staining were obtained from Life Technologies.

### Cell Culture

Cells lines, HL-60 (ATCC-CCL-240), T47D, (ATCC-HTB-133), MCF7 (ATCC-HTB-22), HT-29 (ATCC-HTB-38), HEK-293 (ATCC-CRL-1573), Neuro2A (ATCC-CCL-131), BT549 (ATCC-HTB-122), MDA-MB-231 (ATCC-CRM-HTB-26), and MDA-MB-468 (ATCC-HTB-132), were obtained from American Type Culture Collection (Manassas, VA). All native and transfected cell lines were grown in 50:50 DMEM-F12 media supplemented with penicillin-streptomycin-glutamine mix, non-essential amino acids and 10% fetal calf serum. Stable cell lines expressing wild type MPO were generated using pcDNA3.1 containing the full-length cDNA of human MPO. This construct and the pcDNA3.1 MPO-R569W mutant were kindly provided by William Nauseef, M.D. at the University of Iowa [9]. Following transfection clones were selected with 0.5 mg/ml geneticin (Cellgro) until colonies were visible and could be picked. After colony selection the cell lines were maintained in the presence of 0.2 mg/ml geneticin. It should be noted that selection of T47D stable cell lines was a lengthy process taking up to several months for geneticin resistant colonies to expand to levels necessary for experimentation. Using pcDNA3.1-MPO as a template, MPO cysteine mutants were generated using the Quickchange^™^ site directed mutagenesis kit from Stratagene and cell lines generated as described above. In experiments where cells were treated with catalase for 48 hrs., the media was replaced each 24 hrs with fresh catalase-containing media. In experiments where cells were treated with cathepsin inhibitors, the media was replaced each 24 hrs with fresh inhibitor-containing media. Interestingly, modest levels of cytotoxicity were observed with the cathepsin inhibitors against the T47D-MPO cell line at longer incubation times (~20% cell death at 48 hrs.), however, we found that additional supplementation with catalase (0.5 μM) almost completely negated this cell toxicity. Unfortunately, at effective concentrations the cathepsin inhibitors displayed almost complete cytotoxicity towards other cell lines (HL60, MDA468 and HEK 293) at time points beyond 24 hrs and which could not be abrogated by catalase. In experiments where cells were treated with ammonium chloride and assayed for hexosaminidase or beta-glucuronidase activity, the growth media used was phenol-red free DMEM, penicillin-strep-glutamine mix, non-essential amino acids, 10% heat-inactivated fetal calf serum, 0.2 mg/ml geneticin and 2 μg/ml puromycin (shRNA knockdown cells only). The media was further supplemented with 10 mM ammonium chloride from a 1M, pH 7.4 stock solution as indicated.

### Cell Extracts, Conditioned Media and Partial Purification of MPO on SP Sepharose

One 10 cm culture dish of cells was washed twice with 5 mls Hank’s balanced salt solution with calcium and magnesium (HBSS). 1 ml of ice cold extraction buffer containing 25 mM Tris-HCL pH 7.3, 400 mM KCL, 1% Triton X-100, Complete^™^ protease inhibitor cocktail with EDTA, 1 mM PMSF and 50 nM catalase was added to the dish, cells scraped and pipetted into a 1.5 ml Eppendorf tube. Cells were incubated on ice for 20 minutes and then sonicated with two 10-second pulses using a microtip to create whole cell extracts. Extracts were stored at -80 C or subjected to partial purification depending on the experiment. For HL60 cells which grow in suspension, the cells were pelleted, washed twice by gentle resuspension in HBSS, centrifuged, and the washed cell pellet was resuspended in 1 ml extraction buffer as above. To prepare MPO-containing conditioned media for analysis, the media was removed from cells, brought to 1 mM PMSF and 50 nM catalase followed by centrifuged to remove any cell debris. Conditioned media was then stored at -80°C or subjected to partial purification. Note that catalase is added to extraction buffer or media to remove any H_2_O_2,_ which, while being an MPO substrate, can also inactivate the enzyme. When it was necessary to concentrate MPO and/or remove proteins that created background issues with our analytical techniques, we took advantage of the high positive charge of MPO coupled with cation exchange batch-chromatography to partially purify the enzyme. To do this 1 ml of cell extract was diluted to 7 mls with 10 mM Tris-HCL pH 7.3, 25 mM NaCl, 1 mM EDTA, 0.5% Triton X-100, 20 nM catalase and incubated on a Nutator in a 15 ml polypropylene conical tube at 4 C for at least 4 hrs with 50 μl of SP-sepharose beads. SP-beads were pelleted and washed by resuspending in 5 ml of ice cold 10 mM Tris-HCL pH 7.3, 100 mM NaCl, 0.5% Triton X-100. Beads were then resuspended in 5 ml of room temperature 10 mM Tris-HCL pH 7.3, 200 mM NaCl and incubated for 5 minutes as a more stringent wash. MPO was then eluted with 250 μl of room temperature 10 mM Tris-HCL pH 7.3, 1 M NaCl for 10 minutes and the eluate recovered by centrifugation through a 0.45 um spin-filter (Millipore, UFC30HV00). Eluates were dialyzed against PBS in 10 kDa cutoff microdialysis cups (Thermo-Scientific, 66383) overnight at 4 C. For secreted MPO, 7 ml conditioned media from one 10 cm dish was diluted two fold with 20 mM Tris-HCL pH 7.3, 2 mM EDTA, 0.4% Triton X-100, 50 nM catalase and mixed with 50 μl of SP-sepharose beads for 4 hrs to overnight on a Nutator at 4 C. The subsequent steps were as described for the SP-purification of MPO from cell extracts. Quantitative assessment of wt MPO expression in the T47D cell line was derived from ELISA data on cell extracts combined with a careful cell count. We calculated 1.8 million copies of MPO per cell using a MW of 144 kDa for the MPO heterotetramer. This is an estimate because, unlike the nMPO standard, MPO present in T47D cell extracts is a mixture of proMPO monomer and MPO heterotetramer. A previously published estimate for endogenous MPO in HL60 cells was ~19 (+/-5) million copies per cell [10].

### Immunoblot Analysis of MPO Species

Samples were electrophoresed on NuPage 4–12% Bis-Tris gels in (3-N-morpholino) propanesulfonic acid (MOPS) running buffer as these gels provided for superior resolution of MPO species on non-reducing gels. Samples analyzed under reducing conditions were heated at 95 C for 5 minutes in Laemmli sample buffer with 50 mM dithiothreitol (DTT). Samples analyzed under non-reducing conditions were mixed with Laemmli sample buffer without DTT, incubated at room temperature for 30 minutes and loaded onto the gel without heating. After electrophoresis, proteins were transferred to nitrocellulose overnight in Tris-glycine transfer buffer with 20% methanol. Staining the membrane with Ponceau S protein stain and destaining with 1% acetic acid assessed protein transfer. Membranes were blocked with 4% non-fat dry milk dissolved in PBS + 0.1% TWEEN 20 (PBST). The blot was then probed with HRP-conjugated primary antibody or with unlabeled primary antibody followed by HRP-conjugated secondary antibody and developed with chemiluminescence. In experiments where MPO species are immunoprecipitated prior to immunoblot analysis, 3 μg of Mab-16E3 prebound to 10 μl Ultralink^™^ protein A/G beads (Thermo-Scientific, 53132) was added to each sample diluted to 0.45 ml with immunoprecipitation buffer. The immunoprecipitation dilution buffer was 25 mM Tris-HCL pH 7.3, 150 mM NaCl, 1 mM EDTA, and 1% Triton X-100 and protease inhibitor cocktail. Following immunoprecipitation pellets were washed with modified immunoprecipitation buffer containing 400 mM NaCl and then heated in reducing-Laemmli sample buffer prior to loading onto gels.

### Deglycosylation of MPO with PNGaseF and EndoH

Enzymatic deglycosylation of MPO was carried out in detergent cell extracts, on SP-purified MPO from conditioned media and immunoprecipitated MPO. Prior to glycosidase treatment, MPO in solution or bound to Protein A/G beads was denatured using the buffers supplied with each enzyme and then treated with PNGaseF or EndoH in the presence of protease inhibitors at 37°C for 2 and 6 h respectively. Samples were then analyzed by immunoblot.

### Immunoblot Detection of the Mannose-6-Phosphate (M6P) modification of MPO

The M6P protein modification was detected on immunoblots using a myc-tagged single chain variable domain that binds specifically to M6P (ScFv-M6P). To accomplish this MPO was partially purified from bulk cell extract and conditioned media using SP-sepharose as described above but with the addition of the phosphatase inhibitors sodium fluoride (25 mM), sodium vanadate (1.5 mM) and sodium phosphate (10 mM) to all buffers. The SP-purified MPO was quantitated by ELISA and then a 0.6–0.9 μg equivalent of MPO species was immunoprecipitated with 3.5 μg Mab-16E3. Washed immunoprecipitates were denatured by heating for 5 minutes at 95°C in 10 μl of denaturation buffer (New England Biolabs, B1704) followed by neutralization of SDS with the non-ionic detergent Nonidet P-40. Immunoprecipitates were then treated with or without 10 units of calf intestinal phosphatase in a final volume of 20 μl using the supplied enzyme reaction buffer supplemented with protease inhibitor cocktail without EDTA for 6 hrs at 37°C. calf intestinal phosphatase treatment removes the phosphate group from M6P and disrupts binding by ScFv-M6P on immunoblots, thereby serving as a specificity control. The samples were brought to 1X reducing Laemmli sample buffer, heated again at 95°C, run on 4–12% Bis-Tris gels and transferred to nitrocellulose. Membranes were blocked overnight in TTBS, 4% non-fat dry milk and 50 mM sodium fluoride. After washing in TTBS the membranes were incubated overnight at 4 C with 5–7 μg/ml of ScFv-M6P diluted in TTBS, 1% IgG-depleted BSA and washed three times for 5 minutes in TTBS. Binding of ScFv-M6P was detected by incubating the blot with HRP-conjugated anti-myc tag antibody for 1 h at room temperature followed by chemiluminescent development.

### MPO ELISA and Peroxidase Activity Assay

Quantitation of MPO species was carried out on Costar 96-well ELISA plates (3590) coated for 24–48 hrs at 4 C with 100 μl/well of 0.7 μg/ml rabbit polyclonal MPO antibody (DAKO) diluted in 100 mM carbonate buffer, pH 9.5. Plates were washed with PBST and blocked for 1 h at room temperature with 250 μl/well of 2% non-fat dry milk dissolved in PBST. Samples and a standard curve generated from pure neutrophil MPO (nMPO) were diluted in PBST, 1% IgG-depleted BSA and 100 mM NaCl and incubated on plates (100 μl/well) for 90 minutes at room temperature with gentle agitation on an orbital shaker. Plates were washed with 4 x 250 μl/well of PBST and then incubated with 100 μl/well of HRP-conjugated Mab-16E3 anti-MPO or HRP-conjugated polyclonal goat anti-MPO for 1 hr at room temperature with gentle agitation. Plates were washed again and bound MPO was detected with 100 μl/well of Sureblue^™^ TMB peroxidase substrate mix. After 5–10 min of vigorous shaking the HRP activity was quenched by the addition of 100 μl/well 0.5 M sulfuric acid. Absorbance in each well was determined on a Bio-Tek plate reader at 450 nm. A standard curve was generated using purified neutrophil human MPO (nMPO) serially diluted 1:2 from 31.25 to 0.488 ng/ml. Standard curves were fit by non-linear regression using GraphPad Prism software.

Quantitation of MPO activity was carried out on Nunc^™^ Maxisorp high-capacity 96-well ELISA plates (445101) coated for 24–48 hrs. at 4°C with 100 μl/well of 2 μg/ml rabbit polyclonal MPO antibody (DAKO) diluted in 100 mM carbonate buffer, pH 9.5. Plates were blocked as described above. Samples and standards were diluted as described above (100 μl/well) and plates were incubated for 2 hrs at room temperature or overnight at 4°C. Plates were then washed and the endogenous peroxidase activity of bound MPO was measured by the addition of 100 μl/well of Sureblue^™^ TMB peroxidase substrate mix. After 30–45 minutes of vigorous shaking the reaction was stopped by the addition of 100 μl/well 0.5 M sulfuric acid. The higher binding capacity of the Maxisorp ELISA plates was necessary to immobilize enough MPO to reach a reliable sensitivity range when using the TMB peroxidase substrate. A standard curve for MPO activity was generated by 1:2 serial dilution of nMPO from 250 to 3.91 ng/ml and was included on every 96 well assay plate. Standard curves were fit to the absorbance data as described above. The percent of catalytically active MPO was calculated from the ratio of “active MPO” determined on Maxisorp plates to “total MPO” determined on Costar plates for each sample and then multiplied by 100. For our purpose, it was sufficient to define these values as the “specific activity” relative to the nMPO standard, which was assumed to have a specific activity of 100%. Note that the contribution of the MPO peroxidase activity to the absorbance signal on the Costar plates was insignificant relative to the much higher activity of HRP-conjugated detection antibody. All assay points were conducted in triplicate and at least two preparations of cell extract or SP-purified MPO were tested for each cell line.

### Immunofluorescent Cell Staining

Cells were trypsinized and plated onto uncoated borosilicate glass coverslips. After 48–60 hrs. the cells were washed twice with Hank’s balanced salt solution with calcium and magnesium (HBSS) and fixed for 15 minutes at room temperature in 3% paraformaldehyde in PBS. Cells were then washed once in HBSS, permeabilized for 2 minutes in -20°C methanol, washed twice with HBSS and blocked overnight at 4 C with 5% horse or donkey serum, 20 mM glycine, 0.02% sodium azide diluted in HBSS. Cells were double-labeled with MPO antibody and an organelle marker antibody (as indicated in figures) diluted in 1% IgG-depleted BSA in HBSS for 90 minutes at room temperature. Coverslips were then washed twice with HBSS, 0.25% Triton X-100, once with HBSS and incubated with the appropriate Alexa dye-conjugated secondary antibodies (1:500) diluted in 1% IgG-depleted BSA in HBSS for 1 h at room temperature in the dark. Coverslips were washed as above and in some cases were stained with DAPI nuclear stain diluted in HBSS (0.14 μg/ml) for 5 minutes. Prior to mounting, coverslips were dipped in water to remove salts and set onto a drop of Vectashield mounting media preplaced on slides. Slide quality was initially assessed on a conventional fluorescent microscope (Olympus BX51) and then final images were taken on a Zeiss LSM 710 NLO Multi-photon Laser Point Scanning Confocal Microscope using a 63x oil objective. Though in some figures, images taken using the 100x oil objective on the conventional fluorescent microscope are shown. Images were saved as tiff files in 8-bit per channel format (24-bit RGB) and processed with Photoshop^™^ to produce figures. All images present in this manuscript are representative of at least three independent cell-staining experiments and show the most commonly observed localization for MPO in each cell line.

### Detection of Mannose-6-Phosphate Levels in Cells by Immunofluorescence

Cells were grown on coverslips, fixed and permeabilized as described above. However, instead of blocking the cells in 5% donkey serum, the cells were incubated overnight at 4°C with 5% fetal calf serum. Fetal calf serum contains a high concentration of the soluble form of the cation-independent mannose-6-phosphate receptor (sciMPR) that is shed from the plasma membrane of cells and that retains the M6P binding properties of the full-length receptor [11]. We used binding of the sciMPR to fixed and permeabilized cells to qualitatively assess the total cellular levels of M6P-modified proteins. After binding of the sciMPR, cells were washed and incubated with Mab-86f7 that recognizes only the bovine, but not the human, sciMPR. Binding of Mab-86f7 to bovine sciMPR was visualized with alexa dye-conjugated anti-mouse antibodies. Binding specificity of Mab-86f7 for bovine ciMPR was confirmed because Mab-86f7 did not stain the lysosomes of human T47D cells if they were preincubated with donkey serum instead of fetal calf serum. Specificity was further demonstrated by the ability of free mannose-6-phosphate (5–10 mM) to completely block staining when included in the preincubation with fetal calf serum.

### shRNA Knockdown of cdMPR and SorLA in the T47D-MPO Stable Cell Line

High titer lentiviral preps (3 x 10^8^) of GIPZ shRNA clones and controls were obtained from GE-Dharmacon. The specific clones for cdMPR were V2LHS-234029 and V2LHS-372990 and for SorLA were V2LHS-153328 and V3LHS-322740. Very similar knockdown levels were achieved using either clone for each receptor as judged by immunoblot or quantitative PCR. Briefly, cells were transduced with ~5 virion/cell in growth media supplemented with 8 μg/ml polybrene in a 6-well dish and subjected to spinoculation for 1 h and then left overnight in the incubator. The next morning media was replaced with fresh growth media and cells allowed to recover for 24–48 h. The cells were then selected for 7 days with normal growth media containing 0.2 mg/ml geneticin (MPO selection) and 2 μg/ml puromycin (shRNA selection). After 7 days all cells were visibly expressing green fluorescent protein indicating genomic incorporation of the shRNA vector. Cells were grown with 10 μg/ml puromycin for 3–4 days prior to beginning experiments as, regardless of the ubiquitous GFP expression after selection at 2 μg/ml, we have found that the higher puromycin concentration further improves the level of knockdown in T47D cells as indicated by western blot analysis. During experiments puromycin was returned to 2 μg/ml.

### Quantitative PCR

RNA was isolated using Trizol as described by the manufacturer (Life Technologies, Carlsbad, CA). cDNA was prepared using the High-Capacity cDNA Reverse transcription Kit (Life Technologies). Quantitative PCR was performed using a 7900HT instrument (Applied Biosystems, Foster City, CA). The qPCR was run at 50°C for 2 min, 95°C for 10 min followed by 40 cycles of 95°C for 0.15 min and 60°C for 1 min. The following TaqMan gene expression primers were used (Life Technologies) with TaqMan gene expression master mix (Life Technologies): GAPDH (glyceraldehyde-3-phosphate dehydrogenase), Cat number 4326317E, M6PR (Mannose-6-phosphate receptor), Hs01124299_g1, IGF2R, (insulin-like growth factor 2 receptor), Hs00974474_m1, SORLA, (Sortilin-related receptor), Hs0026342_m1, SORT1, (Sortilin1), Hs00361760_m1, SORCS1 (Sortilin-related VPS10 domain-containing receptor 1), Hs00364666_m1, SORCS2, (Sortilin-related VPS10 domain-containing receptor 2), Hs01019137_m1, SORCS3 (Sortilin-related VPS10 domain-containing receptor 3), Hs01039447_m1. The experiments were repeated three times with the data from a representative experiment shown. The samples were run in duplicate. Data were expressed as the percent of GAPDH, 100*2^-deltaCt^, where deltaCt = Ct_gene of interest_−Ct_GAPDH_. Data were analyzed using GraphPad Prism (GraphPad Software Inc., San Diego, CA).

### Hexosaminidase and Beta-Glucuronidase Assays

Cells were grown in a 12 well dish in duplicate using phenol-red free growth media and heat-inactivated fetal calf serum and supplemented as indicated. Conditioned media (1.4 ml/well) and whole cell detergent extracts (250 μl/well) were then assayed using the fluorogenic substrates 4-Methylumbelliferyl N-acetyl-beta-D-glucosaminide (Sigma, M2133) for hexosaminidase and 4-Methylumbelliferyl-beta-D-glucuronide (Sigma, M9130) for beta-glucuronidase. Conditioned media was assayed undiluted while cell extracts were assayed diluted 1 to 5.6 with extraction buffer so that the enzyme activity of media and extracts could be directly compared volume to volume. Enzyme assays were carried out in triplicate on solid black 96 well plates as such: 16 μl sample + 78 μl 0.1 M acetate pH 4.5 + 6.25 μl of 16 mM substrate (diluted from a 150 mM stock into 0.2% tx-100 in water) per replicate well. Plates were incubated at 37°C for 3 h and then stopped by adding 150 μl of stop solution (33 mM glycine, 83 mM sodium carbonate pH 10.7) and plates were read at excitation/emission of 360/450 nm. Longer time points in pilot experiments showed that product formation in both assays was linear for at least 8 h. The fluorescence measurement (RFU) from corresponding media and cell extract was divided by the protein concentration of the cell extract and finally the duration of the assay and the results were plotted as RFU/hr/mg protein. Normalization to total cell protein mitigates any well-to-well variation in cell number on the tissue culture plate.

## Results

### Processing of pro-myeloperoxidase to the proteolytically cleaved and disulfide-linked mature heterotetramer is observed in two related breast cancer cell lines

**Fig 2:**
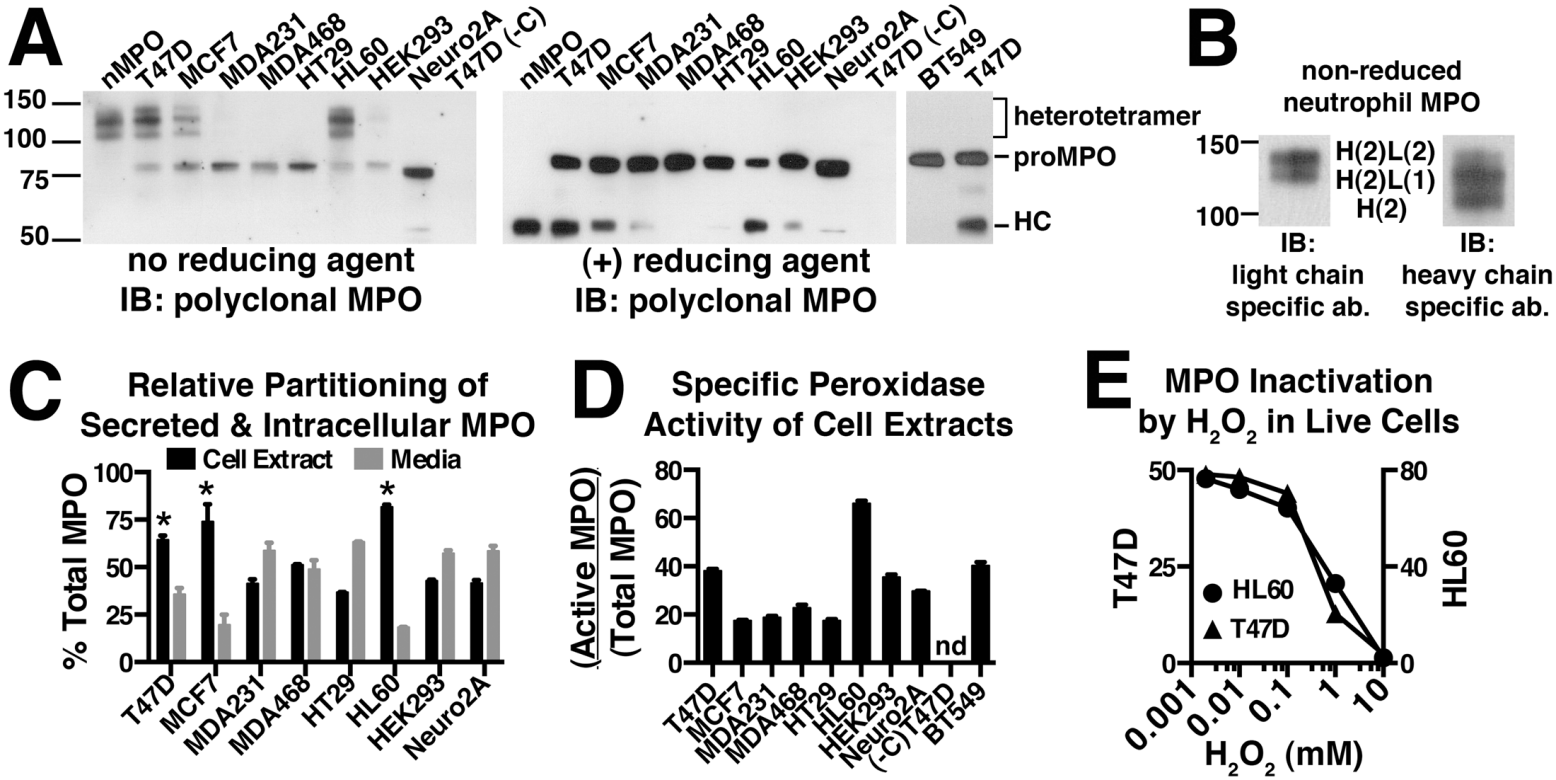
Screening multiple cell lines reveals that T47D and MCF7 cells efficiently process recombinant MPO into the mature heterotetrameric form. (A) Cell extracts were made from the indicated stable cell lines and the total MPO concentration in each extract quantitated by ELISA. Cell extract containing 2 ng of total MPO was run on Bis-Tris gels in the absence or presence of reducing agent and immunoblotted with a multi-epitope polyclonal MPO antibody to assess the MPO species present in each cell line. Under non-reducing conditions the MPO heterotetramer migrates as three bands between the 100–150 kDa markers. Under reducing conditions the heterotetramer falls apart and we instead observe the heavy chain (HC) of MPO that migrates just above the 50 kDa marker. Monomeric proMPO migrates just above the 75 kDa marker and shifts slightly upward upon reduction of the *intra-*molecular disulfide bonds. Untransfected T47D cells (-C) were included as a negative control. (B) The three bands between the 100–150 kDa markers that represent fully processed MPO on non-reducing SDS PAGE were further analyzed to determine if subunit structure was responsible for their distinct gel mobilities. Purified nMPO was run on non-reducing SDS-PAGE and duplicate blots were probed with antibodies specific for either the light chain (L) or heavy chain (H) of MPO. The subunit stoichiometry consistent with the relative immunoreactivity of each band is indicated between the two panels. (C) MPO concentrations in 24 h conditioned media and corresponding whole cell extracts were determined by ELISA and the relative amounts of secreted versus cellular MPO were calculated for each cell line. (D) Whole cell extracts from each cell line were assayed for peroxidase activity and total MPO content and the relative specific activity (Active MPO/Total MPO) determined as described in “Materials and Methods”. (E) Comparison of the sensitivity of native (HL60) and recombinant (T47D) MPO to inactivation by H_2_O_2_. Live cells were treated with increasing concentrations of H_2_O_2_ in the growth media for 1.5 h at which time further inactivation was stopped by the addition of catalase. Specific peroxidase activity was measured in cell extracts generated from treated cells as in panel D. IC_50_ values are 2.8 mM (HL60) and 0.96 mM (T47D-MPO). (C, D & E) Assay points are triplicate measurements and plotted as mean ± SE. The data shown is representative of at least three independent experiments. (nd) None detected.

Differentiated neutrophils do not translate new molecules of MPO and contain only the mature MPO-heterotetramer stored in the azurophil granules. Therefore, studies of MPO biosynthesis have relied heavily on HL60 promyelocytic leukemia cells that, similar to bone marrow-derived promyelocytes, endogenously express, process and store MPO. As such, HL60 cells contain a mixture of the monomeric proMPO, partially processed intermediates and the mature MPO heterotetramer. We sampled cell lines of various tissue origin by stable transfection with MPO in hopes of identifying a recombinant cell model that more closely replicates the biosynthetic characteristics of MPO in promyelocytes and HL60 cells than previously reported expression systems. Our initial screen entailed immunoblot analysis of the MPO present in each cell line to look for the presence of processed MPO species. The MPO heterotetramer (disulfide-linked heterodimers) can be observed intact on SDS-PAGE gels run in the absence of reducing agent or as separate light and heavy chain subunits in the presence of reducing agent. Analysis of cell extracts under non-reducing conditions from each of the recombinant MPO-expressing cell lines revealed that, as expected, all cell lines contained proMPO (Fig 2A, left panel). However, only two related breast cancer cell lines, T47D and MCF7, were able to efficiently process proMPO into the mature heterotetramer and for T47D cells those levels nearly approach the efficiency of processing (ratio of heterotetramer/proMPO on immunoblot) that occur for endogenous MPO in HL60 cells (Fig 2A, left panel). Trace levels of heterotetramer were also visible in MDA231, MDA468 and HEK293 cells when immunoblots were overexposed (not shown). The analysis of cell extracts under reducing conditions shows that the presence of the 55 kDa MPO heavy-chain resulting from proteolytic processing mirrors the relative levels of disulfide-linked heterotetramer in each cell line (Fig 2A, right panel). Additional stable cell lines testing negative for the presence of heterotetramer or heavy chain included SH-SY-5Y and HepG2 cells (not shown). The fact that proteolytic processing and formation of the single *inter-*molecular disulfide bond are not observed separately in any of the recombinant cell lines suggests that these events are mechanistically linked. Among the non-processing cell lines were Neuro2A cells, a neuroblastoma cell line that has been used successfully to study the storage and proteolytic processing of pro-hormones within regulated secretory granules, demonstrating a degree of uniqueness to the protein machinery required for MPO processing [12, 13]. We also included the popular HEK293 cell expression system that has been previously used to characterize MPO processing through biosynthetic radiolabeling [14, 15]. However, comparison of the amounts of processed MPO produced in HEK293 cells to that of T47D and MCF7 cells (Fig 2A) clearly show a significantly greater ability of T47D and MCF7 cells to either process or store the heterotetramer. In cell extracts from T47D and MCF7 cells, we were also able to occasionally observe an ~70 kDa band (see Fig 2A, far right lane), which could represent either minute levels of the MPO processing intermediate or hemi-dimer (heavy and light chain remaining covalently linked together by the heme) resulting from incomplete dissolution of the mature heterotetramer. The MW difference between the previously characterized “70 kDa” processing intermediate that results from cleavage of the pro-domain and the hemi-dimer is only 6 amino acids, a difference too small to distinguish by SDS-PAGE [16].

Mature MPO is a ~144 kDa protein consisting of two heavy chain and two light chain subunits linked together to form a heterotetramer. On non-reducing Tris-glycine gels the heterotetramer migrated as a broad smear (not shown), however, on bis-Tris gels the smear clearly resolved into three bands between the 100 and 150 kDa markers as observed in Fig 2A. We were unclear as to why the heterotetramer would run as three bands considering that each subunit is reportedly covalently linked to another and the fact that under reducing conditions, the 55 kDa heavy chain migrates as a single band. Therefore, we analyzed MPO purified from neutrophils with and without reducing agent using antibodies specific for each of the heavy and light chains (Fig 2B). The results clearly demonstrate that the two lower bands result from loss of one or both of the light chains from the complex. It has been reported for the related eosinophil peroxidase that formation of the covalent linkage between Asp^232^ (Asp^260^ of MPO) and the heme is inefficient and does not occur for all subunits [17]. However, it was also recently reported that when MPO is exposed to its substrate H_2_O_2_, that covalent bonds with the heme are broken and that this leads to increased shedding of the light chain on SDS-PAGE [18]. Whether due to incomplete heme attachment, lability of the heme-protein bonds in the cellular environment or an artifact of SDS-PAGE, the representative banding pattern we observe is the same for MPO heterotetramer derived from either the endogenous or recombinant expression systems.

**Fig 3:**
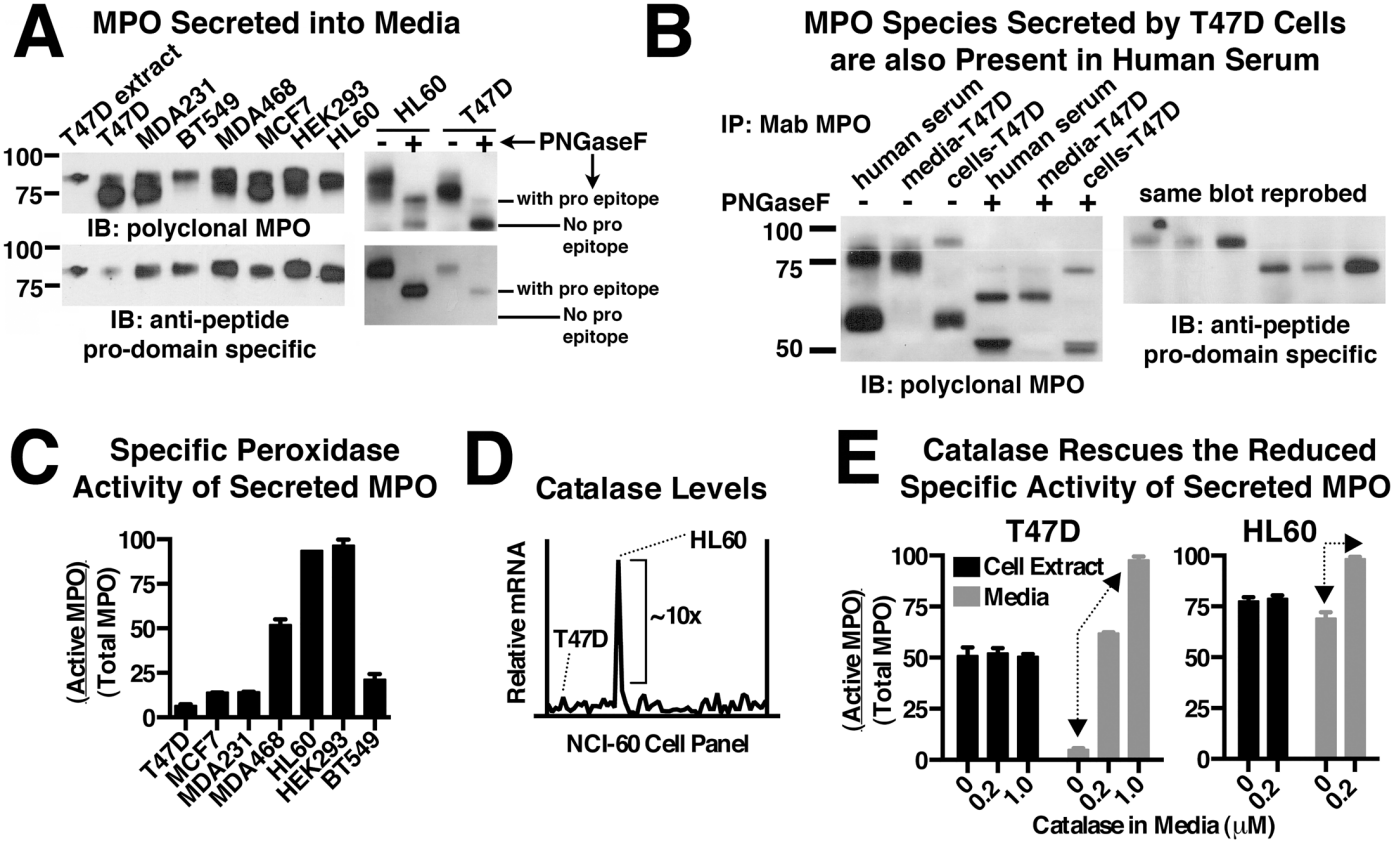
Comparison of constitutively secreted MPO precursors from recombinant cell lines, HL60 cells, and human serum. (A) Secreted MPO species were partially purified from the conditioned media of MPO-expressing cell lines on SP-sepharose and analyzed by immunoblot with a multi-epitope MPO polyclonal antibody (upper blots) or anti-peptide antibody specific for the pro-domain of MPO (lower blots). ProMPO derived from T47D-MPO cell extracts is loaded in the far left lane for comparison (left-hand blots). The nature of the observed immunoreactive bands was further examined by immunoblot analysis of secreted MPO species from HL60 and T47D-MPO cells after complete deglycosylation with PNGaseF to more accurately compare gel mobility and domain specific immunoreactivity (right-hand blots). (B) Immunoblot analysis of MPO species SP-purified and immunoprecipitated from human serum. Three species of MPO are observed in human serum and are defined based on relative mobility and domain-specific immunoreactivity in comparison with cellular and secreted MPO species derived from the T47D-MPO cell line. A reference line is included between glycosylated proMPO and the”cleaved” proMPO intermediate for mobility comparison between left and right blots. The bottom of the right-hand blot is truncated because of strong cross-reactivity of secondary antibodies with the immunoprecipitating antibody in that region. (C) Secreted MPO was partially purified on SP-sepharose and then assayed for peroxidase activity and total MPO content and the relative specific activity (Active MPO/Total MPO) determined as described in “Materials and Methods”. (D) mRNA expression profile of catalase from 59 cancer cell lines plotted in chromatogram format. The data were obtained from the NCI-60 public database and show that HL60 cells express high levels of catalase, exceeding the next highest cell line by a full order of magnitude. (E) Specific peroxidase activity of cellular MPO (cell extract) and secreted MPO (media) derived from HL60 and T47D-MPO cells grown in the absence or presence of catalase for 48 hrs. Arrows indicate the catalase-dependent increase in specific activity observed for secreted MPO, but not cellular MPO, for each cell line. (C & E) Assay points are triplicate measurements and plotted as mean ± SE. The data shown is representative of at least three independent experiments.

For each of the recombinant and endogenous MPO cell lines we were able to detect the presence of MPO in the conditioned media by ELISA. The secreted MPO was partially purified on SP-sepharose and analyzed by immunoblot. In all cases the secreted MPO was detected as overlapping bands in the 73–80 kDa range (Fig 3A, upper-left blot). To determine if differential glycosylation was responsible for the overlapping bands we removed all N-linked oligosaccharides with PNGaseF followed by immunoblot analysis. Representative results are shown for secreted MPO from HL60 and T47D cells (Fig 3A, upper-right blot) and demonstrate that after deglycosylation there are two predominant species present. Furthermore, the protein backbone of each species has an indistinguishable gel mobility whether derived from endogenous or recombinant MPO cell lines. These two MPO species were further defined by their respective ability and inability to be recognized by an anti-peptide antibody specific for the pro-domain of MPO (Fig 3A, lower blots). Together the data suggest the upper band is proMPO and the lower band is a cleaved form of proMPO lacking most of the pro-domain. The “cleaved” proMPO secreted from our cell lines may be equivalent to a previously reported MPO species isolated from HL60 cell media and which was determined to be proteolyzed after K^154^, a site located just upstream from the first amino acid of the light chain (C^167^) [10, 19]. Whether cleavage after K^154^ is unique to secreted MPO or occurs as part of sequential proteolytic cleavage events that are part of normal pro-domain removal during intracellular MPO processing is unknown. The abundance of the cleaved product relative to intact proMPO varied significantly between cell lines and to a lesser extent from prep to prep of conditioned media for the same cell line. Incubation of T47D-MPO conditioned media in the absence of cells overnight at 37°C did not result in further proteolytic cleavage (not shown). The lack of evidence for proteolysis by a secreted protease leaves open the possibility that some fraction of the early intermediate in MPO-processing (the 70 kDa intermediate) gets shunted to the constitutive secretory pathway along with unprocessed proMPO. No significant amounts of the MPO heterotetramer or heavy chain were observed in conditioned media.

One fact that fueled our interest in fully characterizing MPO species secreted from our recombinant cell lines is that MPO precursors have been previously detected in human serum but not in isolated neutrophils [20]. Therefore, we isolated the MPO present in a human serum sample by SP-purification followed by immunoprecipitation and analyzed the MPO-species present by reducing SDS-PAGE and immunoblot (Fig 3B). Three primary species of MPO were identified and included trace levels of proMPO, moderate levels of “cleaved” proMPO and a majority of MPO heterotetramer. Identification of these species in human serum was facilitated by comparison to secreted and cellular MPO species derived from the T47D-MPO cell line. We observed that, after deglycosylation, the protein backbones of the two larger MPO species present in human serum are indistinguishable by gel mobility from those secreted from the T47D-MPO cell line (Fig 3B, left panel). A similar comparison to cellular MPO from the T47D cell line identifies the smallest immunoreactive band in human serum as heavy chain derived from the reduced MPO heterotetramer. Identification of the largest band in human serum as unprocessed proMPO is further indicated by selective immunoreactivity with the pro-domain specific antibody (Fig 3B, right panel). Examination of the MPO species present in four additional human serum samples revealed that these same MPO precursors were present in all samples (not shown). Due to the abundance of circulating neutrophils, the most likely source of the MPO heterotetramer (heavy chain) detected in human serum is neutrophil degranulation. However, the source of the MPO precursors must be promyelocytes, monocytes or other as yet to be described cell types that are actively expressing MPO. MPO levels in human serum have been proposed as a biomarker for several indications including heart disease [21]. If indeed there are distinct cellular sources for the different forms of circulating MPO, antibodies that cross-react with all forms of MPO may be of lower diagnostic value for a given disease state. Furthermore, it may be that both the MPO precursors and the processed MPO heterotetramer possess value as biomarkers but for distinct disease states. For example, one could imagine that high levels of MPO precursors may be diagnostic of promyelocytic leukemia but not cardiovascular disease.

### The cellular and secreted MPO from recombinant cell lines is catalytically active

The level of MPO secreted into the media over a 24-h period and the corresponding cell-associated level of MPO were quantitated by ELISA for each cell line. When calculated as the percent of total MPO (cellular + secreted) it was clear that cell lines that efficiently process MPO into heterotetramer, including T47D, MCF7 and HL60 cells, retain a higher fraction of intracellular MPO (Fig 2C). Since no heterotetramer was observed to be secreted into the media of the T47D and MCF7 stable cell lines, the difference suggested that processed MPO may be stored in an intracellular compartment as occurs in the primary granules of promyelocytes and HL60 cells.

The specific activity of MPO (active MPO/total MPO) was determined by a modified approach related to the recently reported SIEFED technique (specific immunological extraction followed by enzymatic detection) combined with ELISA [22]. They report consecutive determination of both enzyme activity and total MPO in the same well. However, using our substrate and detection antibodies we observed antigen destruction resulting from the MPO activity assay that compromised the subsequent measurement of total MPO. Therefore, we carried out quantitation of MPO activity and total MPO for each sample separately. The specific activity of MPO was determined in cell extracts (Fig 2D) and conditioned media (Fig 3C) for the indicated cell lines. All cell lines contained significant levels of intracellular peroxidase activity, however, the specific activity in HL60 cells was significantly higher than any of the recombinant cell lines. What is perhaps more surprising was that the specific activity of secreted MPO, with the exception of HL60 cells, did not reflect the relative levels observed intracellularly. In fact in T47D-MPO cells which had among the highest specific activity for recombinant cellular MPO had the lowest specific activity for secreted proMPO. We found one possible explanation for this paradoxical observation when we examined mRNA expression levels for catalase that are available from the online NCI-60 database [23, 24]. This database compares several attributes, including mRNA expression profiles of 59 cancer cell lines. Among these cell lines are several that we sampled for MPO processing, including T47D, MCF7, BT549, MDA231, HL60 and HT29 cells. Plotted in chromatogram format, the data show that mRNA levels for catalase in HL60 cells are an order of magnitude higher than the next highest cell line (Fig 3D). MPO is known to undergo irreversible inactivation by its substrate H_2_O_2_ in a concentration dependent manner [18]. There are several cell-based sources of H_2_O_2_ both intracellularly and extracellularly as well as from photochemical reactions involving components of the media itself [25]. The high catalase expression levels in HL60 cells, and likely promyelocytes, has probably evolved to protect MPO from substrate inactivation by the steady state levels of H_2_O_2_ present in actively dividing cells until differentiation of these cells into neutrophils. Indeed, after in vitro differentiation HL60 cells have been shown to downregulate components of the mitochondrial respiratory pathway, which are a major source of reactive oxygen species in cells [26]. To explore this idea we supplemented the growth media of HL60 and T47D-MPO cells with catalase for 48 hours to determine if substrate inactivation was responsible for the loss of MPO activity. Strikingly, we observed an almost complete rescue of the inactive MPO species present in the media of both T47D-MPO and HL60 cells without any significant improvement on the specific activity of intracellular MPO for either (Fig 3E). A similar rescue was observed with secreted MPO activity from the MCF7 and MDA468 stable cell lines (not shown). While the results do not directly address the role of endogenous catalase in the observed specific activities of intracellular MPO, it clearly shows that all secreted MPO is initially active upon exocytosis from the cell prior to undergoing substrate inactivation at levels unique to each cell line. It also suggests that our cell extraction method may inactivate a portion of intracellular MPO prior to the activity assay as otherwise it is difficult to reconcile that all secreted MPO is initially active with the relatively low specific activity measured for the corresponding recombinant cell lines. Alternatively, the different cell lines may incorporate heme into MPO at different efficiencies and, therefore, some percent of what we refer to as intracellular “proMPO” may in fact be “apo-proMPO” and not yet be catalytically active. A final consideration is that HL60 cells and HEK293 cells express MPO at levels an order of magnitude higher than any of the other MPO-expressing cell lines (not shown). Therefore, even if the HL60 and HEK293 cell lines produce similar levels of extracellular H_2_O_2_ as T47D cells, one would expect inactivation of a smaller fraction of the secreted MPO in these cells due to the higher ratio of MPO to H_2_O_2_ [18]

### MPO is stored in the lysosomes of recombinant cell lines that efficiently process proMPO into the heterotetramer

**Fig 4:**
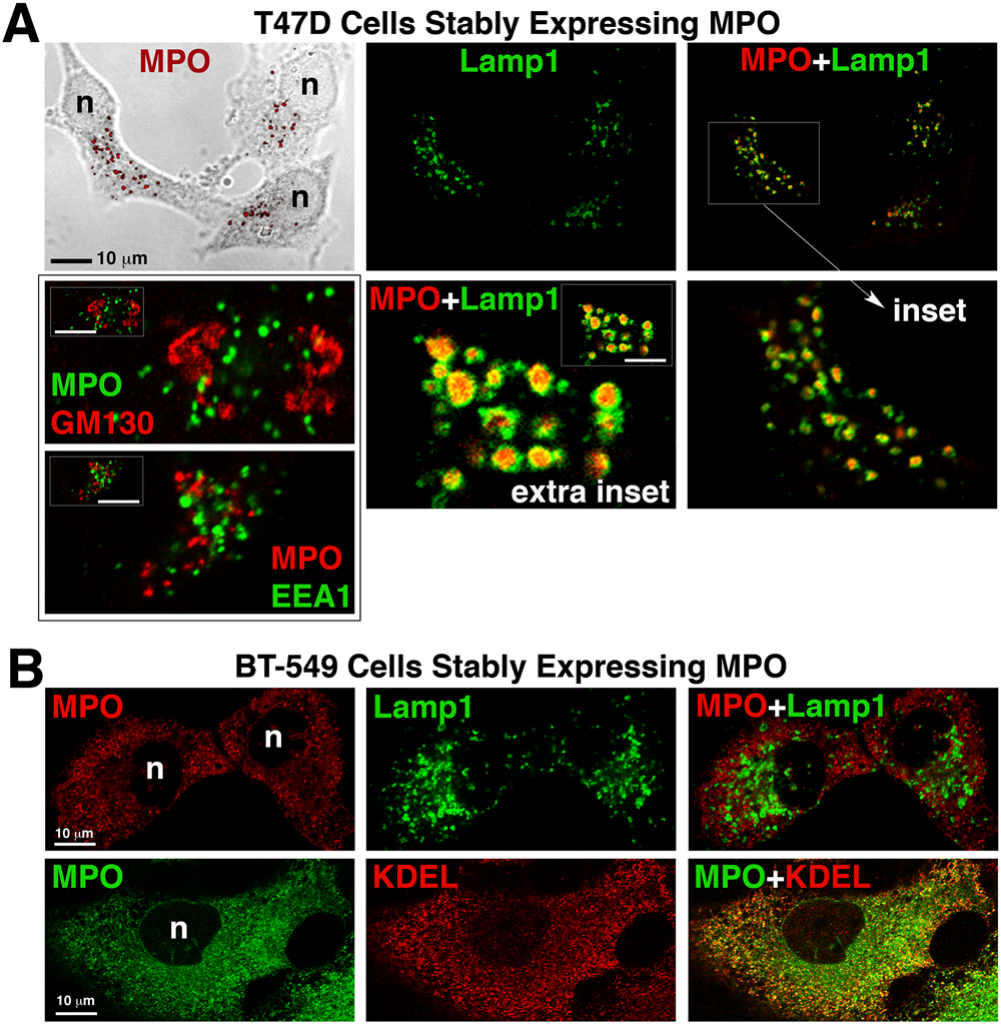
Subcellular localization of MPO in cells that DO (T47D) and DO NOT (BT549) produce heterotetramer. Cells grown on uncoated glass coverslips were fixed, permeabolized and double-labeled with antibodies against the indicated proteins. Mounted coverslips were imaged with a 63x oil objective on a Zeiss LSM 710 confocal microscope. (A) Fluorescent images of the T47D-MPO stable cell line showing that MPO colocalizes with the lysosomal membrane protein Lamp1, but not with the early endosome protein EEA1 or the cis-Golgi protein GM130. An extra inset of a cell with very large lysosomes is included to more easily visualize that MPO-immunoreactivity (red) is concentrated within the lumen of the lysosomes. (B) Fluorescent images of the BT549-MPO stable cell line showing that MPO does not colocalize with the lysosome marker Lamp1 in these cells, but, the punctate staining pattern does partially co-localize with the endoplasmic reticulum marker KDEL. (n) nucleus.

In promyelocytes and HL60 cells the processed MPO heterotetramer, but not proMPO, is stored in azurophil granules that are a type of secretory lysosome. We had determined through biochemical experiments that the MPO heterotetramer accumulated intracellularly in recombinant T47D and MCF7 cell lines. Therefore, we immunolocalized MPO in these cell lines as well as in the non-processing cell lines to determine its subcellular localization. In confocal images of T47D-MPO cells the most prominent staining for MPO is in punctate granules where it colocalizes with the lysosomal marker Lamp1, but not with the early endosome marker, early endosome antigen 1 (EEA1) or with the Golgi marker, golgin subfamily A member 2 (GM130) (Fig 4A). More precisely, MPO staining was localized to the lumen of the lysosome where it was encapsulated by Lamp1 (Fig 4A insets). While more difficult to visualize next to the bright punctate staining, MPO staining also colocalized with a marker for the endoplasmic reticulum (ER) in these cells (S1A Fig, arrowhead). MPO in MCF7 cells shows a similar localization pattern to that of T47D cells (S1B Fig). In cell lines that did not significantly process MPO such as BT549, MDA468 and Neuro2A cells, no prominent MPO staining was present in lysosomes but instead was diffuse and colocalized most significantly with the ER marker (Fig 4B and S1C & S1D Fig) and is similar to what has been previously reported for MPO expressed in BHK cells [27]. The anti-peptide antibody specific for the pro-domain of MPO (used in Fig 3A & 3B) only worked for immunoblotting and we were unable to directly contrast the subcellular localization of proMPO with that of the heterotetramer using a combination of our MPO antibodies. Nonetheless, given the totality of the results and in context with the literature we would assume that ER-localized MPO is either proMPO or apo-proMPO whereas the immunoreactive MPO localized to lysosomes is the mature heterotetramer. The observation of significant ER-resident MPO in all cell lines is qualitatively consistent with previously reported electron microscopy in HL60 cells and pulse-chase experiments in both endogenous and recombinant MPO-expressing cells showing that newly translated MPO spends a prolonged period in the ER prior to entering the Golgi for mechanistic reasons that are not yet fully explained [4, 28, 29]. The fact that no significant MPO staining was observed in the Golgi or in early endosomes suggests that MPO traffics rapidly through these vesicular structures on the way to either the lysosome or secretion into the media.

After confirming storage of MPO in the lysosomes of T47D-MPO cells we wanted to quantitatively compare the substrate inhibition of endogenous (HL60) and recombinant (T47D) MPO by H_2_O_2_. H_2_O_2_ is permeable through certain aquaporins in the plasma membrane, however, substrate or potential inhibitors would also have to cross a second membrane to access MPO stored in lysosome granules [30]. When live HL60 or T47D-MPO cells are presented with a dose-escalating bolus of H_2_O_2_ we observed that intracellular MPO peroxidase activity is decreased in a dose-dependent manner and is completely lost at the highest concentration of H_2_O_2_ (Fig 2E). Comparison of the dose-response curves reveals a slightly lower H_2_O_2_-sensitivity for MPO in HL60 cells possibly owing to the high catalase expression levels in these cells, but nothing of the magnitude that would suggest a difference in the attachment or chemistry of the heme prosthetic group in recombinant versus endogenously expressed MPO. Nor was either curve biphasic, which may have suggested a substantial difference in the accessibility of H_2_O_2_ to ER-localized proMPO versus the heterotetramer present inside lysosomes. We do not know what fraction of intracellular proMPO present in HL60 and T47D-MPO cells is catalytically active but base our assumption of significant activity on the substantial peroxidase activity observed in non-processing cell lines such as BT549 that contain only proMPO. The results indicate that H_2_O_2_ is able to readily cross cell membranes to access MPO in these cell lines. At lower, naturally occurring concentrations of H_2_O_2_ one might expect variations in catalase expression to significantly alter such access in a cell-specific manner. The accessibility of H_2_O_2_ to MPO stored intracellularly is a necessary prerequisite for high-throughput screening of cell-permeant MPO inhibitors, a task for which the technical hurdles are greatly simplified by having the flexibility of screening against cells that grow both in suspension (HL60, neutrophils) and attached to a substrate (T47D-MPO) depending on the desired pharmacological parameter to be measured.

### Comparison of N-glycosylation and modification with mannose-6-phosphate of MPO produced by recombinant cell lines

**Fig 5:**
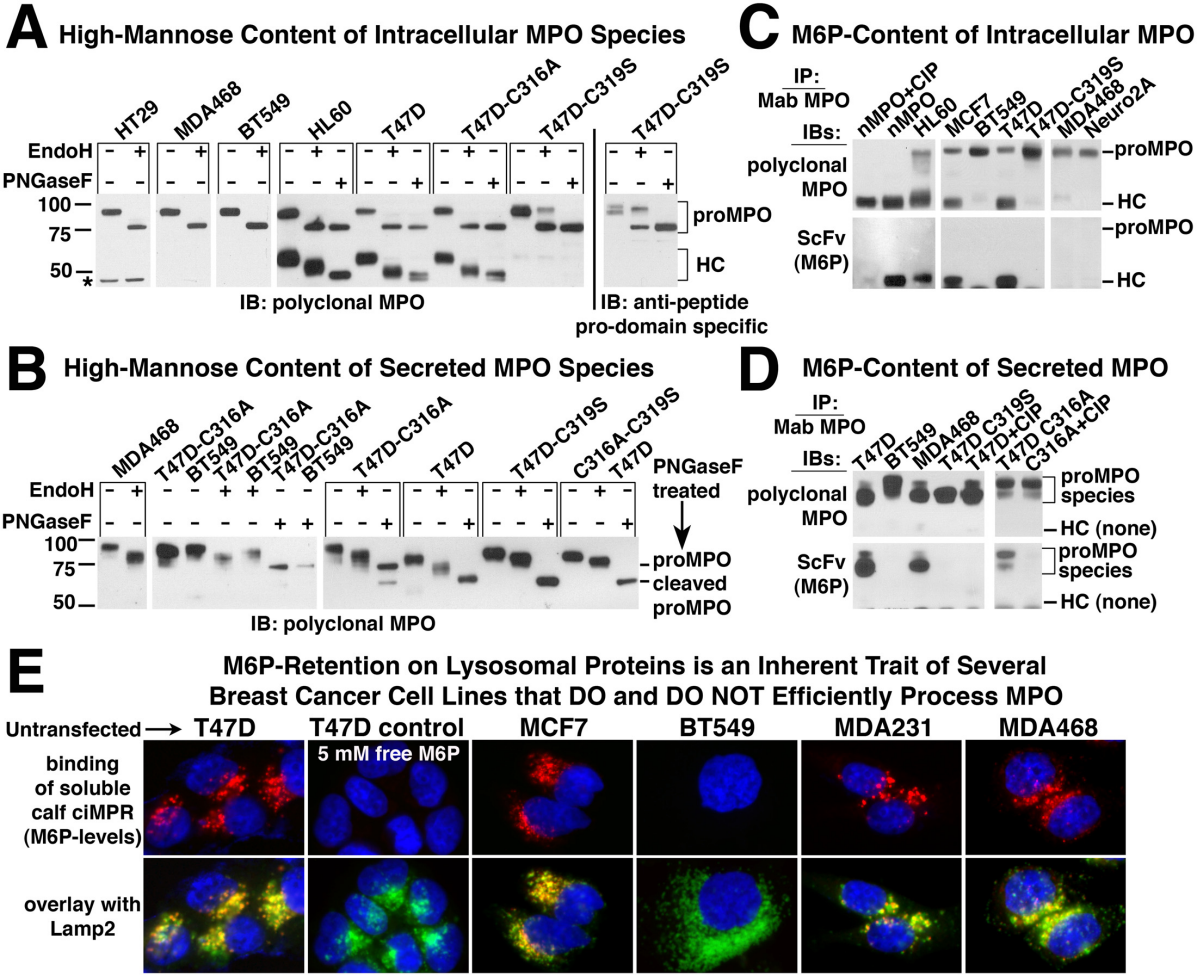
Characterization of N-Glycosylation and Mannose-6-Phosphate modification of wildtype and mutant MPO from recombinant cell lines. (A) Cell extracts from the indicated endogenous (HL60) or recombinant MPO-expressing cell lines were treated with the glycosidases EndoH or PNGaseF as indicated and analyzed for alterations in relative mobility by immunoblot (see Materials and Methods). The four left-hand panels were probed with polyclonal multi-epitope MPO antibody and the far right panel with anti-peptide antibody specific for the “pro” domain of MPO. The results in the right-hand panel aid in the visualization of the change in glycosylation pattern observed with the C319S MPO mutant as the pro-epitope is unaffected by glycosylation pattern whereas recognition by the multi-epitope polyclonal antibody is negatively affected by the increased complex oligosaccharide content of this mutant. *Non-specific band in HT29 extracts. (B) Secreted MPO was partially purified on SP-sepharose from the conditioned media of MPO-expressing cell lines, treated with the glycosidases EndoH or PNGaseF as indicated and analyzed by immunoblot as in panel A. Of the two secreted MPO species the full-length proMPO was predominant in MDA468, BT549 and T47D-C316A cells whereas proMPO with the pro domain proteolytically removed (“cleaved” proMPO) was the predominant form observed in T47D, T47D-C319S and T47D-C316AC319S cells. (C) Eight hundred ng of MPO partially purified on SP-sepharose from native (HL60) and recombinant cells extracts or 500 ng of pure neutrophil MPO was immunoprecipitated with the Mab-16E3 MPO antibody. Immunoprecipitates were analyzed by immunoblot under reducing conditions with multi-epitope polyclonal MPO antibody to detect all MPO species (upper blots) or with a single chain variable domain (ScFv) specific for the mannose-6-phosphate (M6P) modification (lower blots). (D) Eight hundred ng of secreted MPO partially purified on SP-sepharose from the indicated cell lines was immunoprecipitated with Mab-16E3 MPO antibody. Immunoprecipitates were analyzed by immunoblot as in panel C. (C & D) The specificity of the ScFv for M6P recognition is demonstrated by the loss of signal when immunoprecipitated MPO is treated with calf intestinal phosphatase (CIP). Faint bands on the bottom of ScFv blots are background recognition of the immunoprecipitating antibody that migrates just below the MPO heavy chain on SDS-PAGE. (E) Untransfected T47D, MCF7, BT547, MDA231 and MDA468 cells, were fixed, permeabilized and stained for total cellular M6P levels using the soluble form of bovine cation-independent mannose-6-phosphate receptor (sciMPR). Binding of the sciMPR was detected with Mab-86f7, which binds to bovine, but not human ciMPR. Lysosomes are stained with antibodies against Lamp2. Competitive inhibition of sciMPR binding with 5 mM free M6P in T47D cells was included as a specificity control.

Glycosylation patterns of both mature MPO from neutrophils and recombinant proMPO secreted by Chinese hamster ovary cells (CHO) have been characterized in detail [31, 32]. All MPO glycosylation is of the N-linked type and of the six confirmed glycosylation sites, five lie within the boundaries of the heavy chain and one is present in the pro-domain. To determine if there were any clear correlation between the ability of a cell line to process MPO and the nature of the glycosylation pattern, we examined these modifications by treating select cell extracts or secreted MPO with EndoH for removal of high mannose oligosaccharides or PNGaseF for complete deglycosylation and analyzed the results by immunoblot under reducing conditions. In cell extracts from recombinant and endogenous cell lines, whether capable of processing MPO or not, all glycosylation of proMPO is of the high-mannose type with no discernible difference in gel mobility between EndoH and PNGaseF treated samples. This pattern is expected for a protein that has not yet exited the ER. Comparison of gel mobility for the processed heavy chain in HL60 and T47D-MPO cells shows that a mixture of high mannose and complex oligosaccharides are present (Fig 5A). There appears to be more high-mannose modification of heavy chain from T47D cells than in HL60 cells indicated by a larger mobility shift upon EndoH treatment. However, the difference does not appear to significantly impact MPO processing. We also observed that after complete deglycosylation with PNGaseF, the MPO heavy chain from HL60 cells migrates as a single band, whereas that from T47D (Fig 5A) and MCF7 (not shown) cells is a doublet. Whether the doublet is representative of the previously reported I, II and III isoforms of MPO which are separable by ion exchange chromatography and are reportedly caused by as of yet unidentified modifications of heavy chain or slightly altered proteolytic processing is unclear [33]. It is possible that the convertase(s) involved in that particular cleavage event in T47D or MCF7 cells are related to but distinct isoforms from that found in HL60 cells. Analysis of secreted proMPO species was more difficult because of the presence of both proMPO and its cleaved product migrating in close proximity to one another on the gel. However, when secreted MPO from T47D, BT549 and MDA468 cells was analyzed for gel mobility shift after glycosidase treatment each retained some level of high mannose oligosaccharide with BT549 containing the least (Fig 5B).

Modification of high mannose oligosaccharides with mannose-6-phosphate (M6P) occurs for endogenous MPO and this modification is retained on the mature heterotetramer [34–36]. We wanted to measure the extent of this modification in recombinant MPO as another gauge of how similar the processing events are between the endogenous and recombinant cell systems. Therefore, we immunoprecipitated cellular and secreted MPO species and assayed for the presence of M6P by immunoblotting with a recombinant single chain variable domain (ScFv) specific for this modification [8]. Of the processing and non-processing cell lines analyzed, only those with processed MPO (HL60, MCF7 & T47D), indicated by the presence of heavy chain, had detectable levels of M6P (Fig 5C). No cell line showed labeling of intracellular proMPO, consistent with the bulk of proMPO not yet having entered the Golgi where enzymes responsible for the two-step M6P modification reside. Specificity of the ScFV for M6P was demonstrated by a lack of immunoreactivity of neutrophil MPO after treatment with calf intestinal phosphatase.

Since no M6P was detected on intracellular proMPO using the ScFV, but intracellular proMPO in HL60 cells has been observed to incorporate radioactive inorganic phosphate [36], we examined secreted MPO from cells that do and do not efficiently process MPO for the presence of M6P. Unlike intracellular proMPO, secreted material has both complex and high-mannose oligosaccharides, demonstrating that it has passed through the Golgi and been potentially exposed to phosphotransferase. Analysis of secreted MPO species from T47D, MDA468 and BT549 cells shows that both intact proMPO and the cleaved species possess M6P when derived from T47D and MDA468 cells but not from BT549 cells (Fig 5D). Since the MDA468 cell line does not efficiently process MPO, this suggests that the presence of M6P, while potentially being necessary for MPO processing, does not appear to be sufficient.

In most cells types the M6P modification of enzymes destined for transport to the lysosome does not accumulate and is rapidly removed by lysosomal acid phosphatase 2 and tartrate-resistant acid phosphatase after transport to the late endosome/lysosome [37, 38]. However, in select cell types such as neurons the M6P modification is retained on enzymes stored in the lysosome [39]. To determine whether the retention of the M6P modification was specific to processed MPO in T47D and MCF7 cells or whether it is a general property of other enzymes stored in the lysosomes of these cells, we stained untransfected T47D and MCF7 cells for M6P content using the soluble fragment of the cation-independent mannose-6-phosphate receptor (sciMPR) and compared the staining to that of breast cancer cell lines in which no significant processing of MPO was observed. Detection of bovine sciMPR binding in these experiments was accomplished using a monoclonal antibody that binds only to bovine ciMPR, but not to human ciMPR [7]. With the exception of BT549 cells, bright staining of lysosomes for M6P was observed in all cell lines regardless of the ability to efficiently process MPO (Fig 5E). Staining specificity was demonstrated in T47D cells (control) by competitive inhibition of sciMPR binding with 5 mM of free M6P, showing that binding of the sciMPR is specific for M6P-modified proteins and that Mab-86f7 does not bind to the endogenous human ciMPR. The results indicate that M6P-retention is a general trait of lysosomal enzymes in T47D and MCF7 cells rather than being specific to MPO expressed recombinantly in these cells. The fact that the non-processing cell lines MDA231 and MDA468 share this trait further suggests that M6P-modification and/or retention is not a common limiting factor in the inability of most cell types to process and store MPO. However, a requirement for the M6P-modification cannot be ruled out. Consistent with this possibility, we clearly observed trace levels of the MPO heterotetramer in cell extracts derived from the MDA231 and MDA468 stable cell lines when immunoblots were overexposed whereas none could be detected in extracts derived from the BT549 cell line when analyzed on the same blot (not shown). Whether retention of the M6P moiety is a trait of normal breast epithelial cells or occurs only in select breast cancer cell lines is beyond the scope of our research. However, significantly enhanced levels of M6P-modified proteins have been reported in breast tumors [35].

### Proteolytic separation of the heavy and light chains of MPO is a separable event from formation of the *inter-*molecular disulfide bond

**Fig 6:**
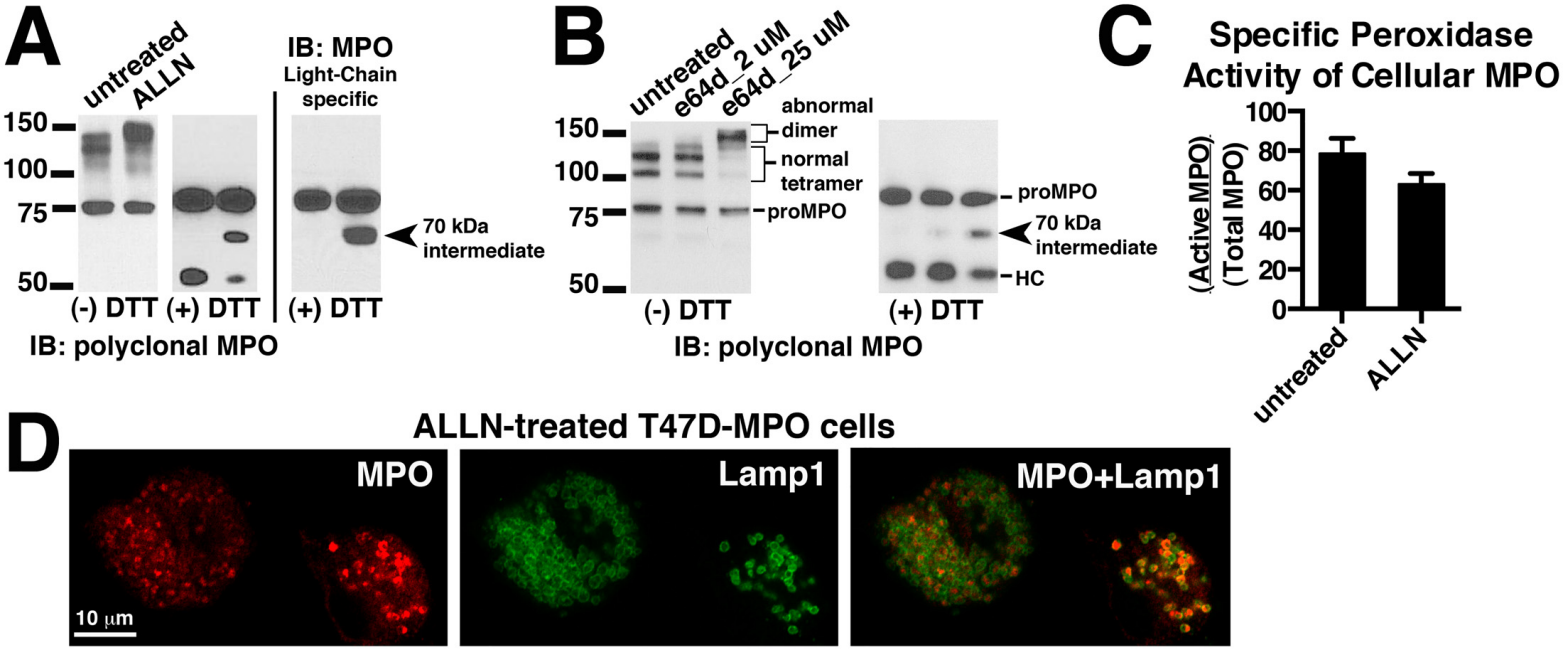
Effect of cathepsin inhibitors on the processing, activity and endocytic trafficking in T47D-MPO cells. (A) T47D-MPO cells were grown with or without 8 μM ALLN for 44 h. MPO was partially purified from the resulting cell extracts on SP-sepharose and analyzed by immunoblot under non-reducing (-DTT) and reducing (+DTT) conditions with multi-epitope polyclonal MPO antibody to detect all MPO species (left panel) or antibodies specific for epitopes within the light chain of MPO (right panel). (B) T47D-MPO cells were grown in the presence of the indicated concentrations of e64d for 44 h and the resulting cell extracts analyzed by immunoblot under non-reducing (-DTT) and reducing (+DTT) conditions with multi-epitope polyclonal MPO antibody. (C) Samples analyzed in panel A were assayed for peroxidase activity and total MPO content and the relative specific activity (Active MPO/Total MPO) determined as described in “Methods and Materials”. (D) T47D-MPO cells were grown on glass coverslips in the presence of 8 μM ALLN for 72 h. The treated cells were fixed, permeabolized and double-labeled with antibodies against MPO (red) and Lamp1 (green). Mounted coverslips were imaged with a 63x oil objective on a Zeiss LSM 710 confocal microscope.

It has been previously shown that cathepsin (cysteine protease family) inhibitors block the cleavage events that lead to the excision of a hexapeptide from MPO and results in the separation of the heavy and light chains [15]. However, possibly because of the limitations of the cell models used, it was not determined how the inhibition of this proteolytic event affected other aspects of MPO processing such as inter-molecular disulfide bond formation or on the trafficking and storage of MPO in lysosomes. Using our T47D cell expression system we were able to build upon these initial results. We treated T47D-MPO cells for ~48 hours with the indicated concentrations of the cathepsin inhibitors ALLN or e64d and analyzed MPO species in the resulting cell extracts by immunoblot under reducing and non-reducing conditions. Under reducing conditions we observed a decrease in the amount of heavy chain and the concomitant accumulation of the 70 kDa processing intermediate, the identity of which was inferred by its gel mobility combined with its recognition by light chain-specific antibodies (Fig 6A middle and right blots, Fig 6B right blot). There was no effect on the level of proMPO. Under non-reducing conditions we observed that the immunoreactive bands representing the normal heterotetramer decrease after treatment with either inhibitor, and that a larger ~150 kDa band appears (Fig 6A left blot, Fig 6B left blot). This suggests that the uncleaved 70 kDa intermediate can be efficiently disulfide bonded to either another 70 kDa intermediate or a normally cleaved hemi-dimer and, therefore, the heterotetramer is replaced by abnormal trimer and dimer depending on the efficiency of protease inhibition. We can infer that this is occurring because of the appropriate increase in the gel mobility and an almost complete loss of light chain shedding that results in the three-band pattern observed for the normal heterotetramer on non-reducing gels. We also measured the specific peroxidase activity of MPO derived from ALLN-treated cell extracts and found it was only modestly reduced versus untreated cells (Fig 6C). Since in our cell line screen we never observed proteolytic separation of heavy and light chains in the absence of the *inter-*molecular disulfide bond or vise versa (Fig 2A), this suggested a mechanistic interdependence of these events. Therefore, the results with the cathepsin inhibitors suggest that the *inter-*molecular disulfide bond is formed prior to proteolytic separation of the heavy and light chains. As a byproduct of the ALLN/e64d treatments we are also able to approximate the turnover rate of MPO heterotetramer in T47D cells. Whether MPO in lysosomes is specifically degraded or whether it is simply subjected to degradation as a consequence of lysosome turnover is unknown. Ideally we would have liked to replicate these observations for endogenously expressed MPO in HL60 cells, however, effective concentrations of the inhibitors caused extensive cell death in HL60 cells and we were unable to obtain meaningful results. Indeed we found the robustness of the T47D cell line to several cytotoxic agents a valuable attribute of this expression system.

We next asked whether blocking separation of heavy and light chains would effect lysosomal transport of MPO and perhaps trap the abnormal dimer in whatever intermediate endosomes in which these processing events occur. To do this we immunolocalized MPO in T47D-MPO cells treated with ALLN for 72 hours to ensure almost complete turnover of heterotetramer to the abnormal dimer. The lysosomes were morphologically altered by the treatment and appeared swollen, nonetheless, all prominent MPO staining was encapsulated within the staining of the lysosomal membrane marker Lamp1 (Fig 6D). This suggests that separation of the heavy and light chains is not necessary for trafficking of MPO to the lysosome. It will be interesting in the future to determine whether proteolytic removal of the pro-domain is equally dispensable for formation of the *inter-*molecular disulfide bond or lysosomal transport. For example, proteolytic removal of the pro-domain may serve as the passkey which sets MPO on the path to the lysosome, diverting it from the constitutive secretory pathway, or it may simply occur as a byproduct of MPO associating with a yet to be discovered endosomal receptor that directs it through the correct endocytic pathway. Simple deletion of the pro-domain resulted in decreased secretion, ER-retention and proteosome-mediated degradation of intracellular MPO expressed in K562 or PLB-985 cells, therefore, a more subtle experimental approach will be necessary to dissect its role in the post-ER processing events of MPO [40, 41].

### Probing the importance of the *inter*-molecular disulfide bond of MPO through expression of cysteine mutants in the T47D cell system

**Fig 7:**
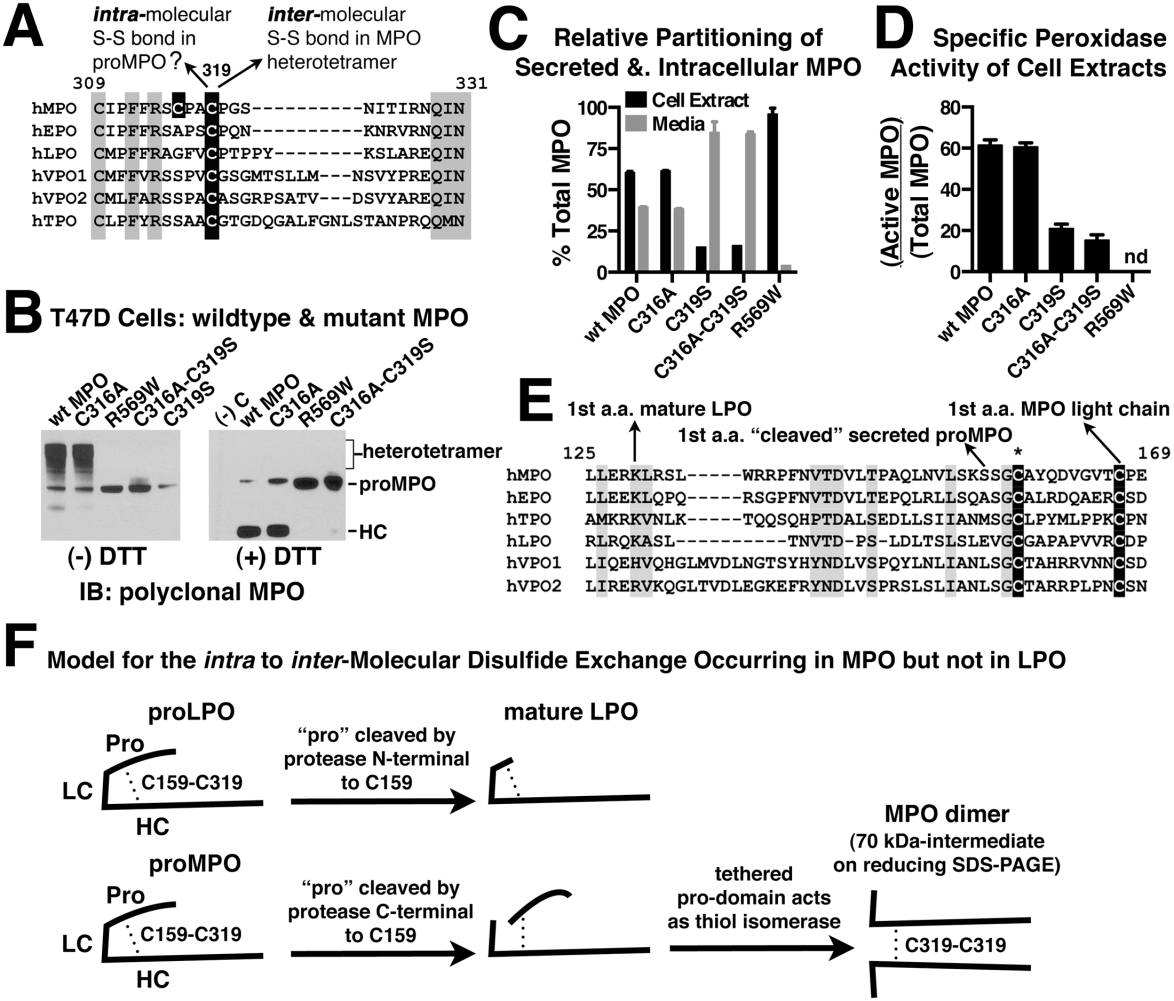
Mutation of Cys-319, but not Cys-316, disrupts MPO-folding exposing a role preceding *inter*-molecular disulfide bonding. (A) Sequence alignment of human MPO with other members of the human peroxidase family shows the relative conservation of mutated cysteine residues (black shading). Alignment numbering starts at the first amino acid in the signal peptide of unprocessed MPO. (B) Whole cell extracts from T47D cells stably expressing wildtype (wt) and mutant MPO were analyzed by immunoblot under non-reducing (left blot) and reducing (right blot) conditions to assess the effect of mutations on MPO processing. Untransfected T47D cell extract was included as a negative control (right blot, (-)C). (C) The concentration of wt or mutant MPO present in 24 h conditioned media and corresponding cell extracts was quantitated by ELISA and the relative amounts of secreted versus cellular MPO were calculated for the indicated T47D cell lines. (D) Cell extracts derived from the indicated T47D stable cell lines were assayed for peroxidase activity and total MPO content and the relative specific activity (Active MPO/Total MPO) determined as described in “Methods and Materials”. (C & D) Assay points are triplicate measurements and plotted as mean ± SE. The data shown is representative of at least three independent experiments. (nd) None detected. (E) Sequence alignment of the C-terminal portion of the pro-domain of human peroxidase family members. Conserved cysteines are shaded in black. (*) Cys 158, which forms the *intra*-molecular disulfide bond with Cys 319 observed in the lactoperoxidase (LPO) crystal structure. Arrows pointing at the first residue of the mature proteins indicate final pro-domain cleavage sites for processed MPO and LPO. The cleavage site for the truncated proMPO species previously isolated from HL60 cell media [19] is also indicated with an arrow (“cleaved” secreted proMPO). (F) Diagram contrasting how small changes in the proteolytic processing of MPO versus LPO in the region surrounding Cysteine-159 may have evolved to facilitate the later use of Cysteine-319 for MPO’s unique inter-molecular disulfide bond.

Because of the combination of high efficiency MPO processing and the fact that these are reasonably large and easily imaged cells, T47D cells allow for complementary studies that both examine MPO processing via a biochemical approach and MPO trafficking via immunofluorescence. We took advantage of these attributes by creating mutants to further probe the importance of the *inter-*molecular disulfide bond of MPO. The MPO heavy chain has 12 cysteine residues 10 of which form five *intra-*molecular disulfide bonds within the endoplasmic reticulum [5]. One of the two remaining cysteines (Cys^319^) is utilized at a later stage of processing to form the *inter-*molecular disulfide bond with its counterpart to link together the two heavy chains in the MPO heterotetramer. The spatio-temporal details of *inter-*molecular disulfide bonding are unknown and may occur in the trans-Golgi, endosomes or possibly upon arriving to the final destination in the lysosome granule. Cys^319^ is conserved in all members of the human peroxidase family, though only MPO has been shown to form an *inter-*molecular disulfide bond using this residue (Fig 7A) [42, 43]. We mutated Cys^319^ to a serine residue. Interestingly, the remaining cysteine in the MPO heavy chain is Cys^316^ and is conserved in all known orthologs of MPO but not in other family members. Together these two cysteines resemble a CXXC motif which is found in the active site of many redox proteins that participate in the formation, isomerization, and reduction of disulfide bonds [44]. We mutated Cys^316^ to alanine (the corresponding residue in EPO) to examine any potential role in catalyzing the formation of MPO’s unique *inter-*molecular disulfide bond. Stable lines were generated for each single cysteine mutant and a double cysteine mutant in T47D cells and then examined for the effects on MPO processing, activity and endocytic trafficking. As an additional control we also examined the R^569^ to W missense mutation, an inactivating mutation that occurs in a subset of patients suffering from myeloperoxidase deficiency and which has been previously characterized as a recombinant protein in K562 cells [9].

Cell extracts from wildtype (wt) and mutant MPO cell lines were analyzed by immunoblot both in the absence and presence of reducing agent to assess MPO processing. The C316A mutant undergoes normal processing events indicated by levels of heterotetramer (-DTT) and cleaved heavy chain (+DTT) that is identical to those produced in wt T47D-MPO cells (Fig 7B). Both the C319S and double cysteine mutants display severely decreased proteolytic processing and, as expected, do not form an *inter-*molecular disulfide bond. At the level of immunoblot the R569W mutant resembles the C319S and double cysteine mutants in its lack of processing.

ELISA measurements of cellular versus secreted MPO from wt and mutant cell lines show that the C316A mutant again behaved similarly to wt MPO. Both the C319S and double cysteine mutants demonstrated enhanced secretion from cells while secretion of the R569W mutant was severely decreased (Fig 7C). The opposing effect of the C319S and R569W mutations on the secretion of MPO suggested that, while both disrupt MPO processing, they have distinct effects on MPO trafficking. Peroxidase activity measurements of cellular MPO show that the C316A mutant has catalytic activity comparable to wt MPO and the C319S and double mutants have decreased specific activity that is 1/4 to 1/3 of that from wt MPO cells (Fig 7D). The decreased activity suggests that either heme attachment or conformational stability was partially compromised in the C319S and double cysteine mutants. Peroxidase activity for the R569W mutant in cell extracts was undetectable in our assay.

Glycosylation characteristics including modification with M6P were examined for the cysteine mutants. All such modifications of the C316A mutant were similar to what was observed for wt MPO in T47D cells (Fig 5A–5D). For the non-processed C319S and double cysteine mutant we observed significant differences from wt MPO in both glycosylation and M6P content. EndoH treatment of C319S cell extracts resulted in two visible species of proMPO one containing high-mannose oligosaccharides similar to wt MPO and a second novel band containing primarily complex oligosaccharides. Both species collapse into a single band representing the proMPO backbone when completely deglycosylated with PNGaseF (Fig 5A right side). It also appears that the high content of complex oligosaccharides in C319S may negatively impact recognition by the polyclonal MPO antibody when compared to recognition by the anti-peptide pro-domain specific antibodies (Fig 5A right side). The anti-peptide antibodies should be less sensitive to glycosylation effects on immunoblot and suggest a more equal level of the two-glycosylated species. No M6P content was detected in either of the intracellular proMPO species from C319S cells (Fig 5C). Secreted MPO from both the C319S and C316A-C319S cell lines displayed an increase in the content of complex oligosaccharides relative to MPO secreted from the wt MPO-T47D cells and is consistent to what was observed for intracellular MPO from C319S cells (Fig 5B, right panel). The increased content of complex oligosaccharides on secreted MPO from the non-processed cysteine mutants is similar to previous observations of secreted MPO species obtained from 32D cells expressing a pro-domain deletion mutant and together with our data suggests that proper glycosylation requires specific structural cues [40]. Nonetheless, a small shift in gel mobility upon EndoH treatment is evident for secreted MPO from the non-processed cysteine-mutant cell lines indicating the presence of high-mannose oligosaccharide on at least one N-linked site and leaving the possibility for modification of secreted MPO by M6P (Fig 5B). However, unlike wt MPO and the C316A mutant, no M6P was detected on secreted MPO derived from the C319S mutant cell line (Fig 5D).

**Fig 8:**
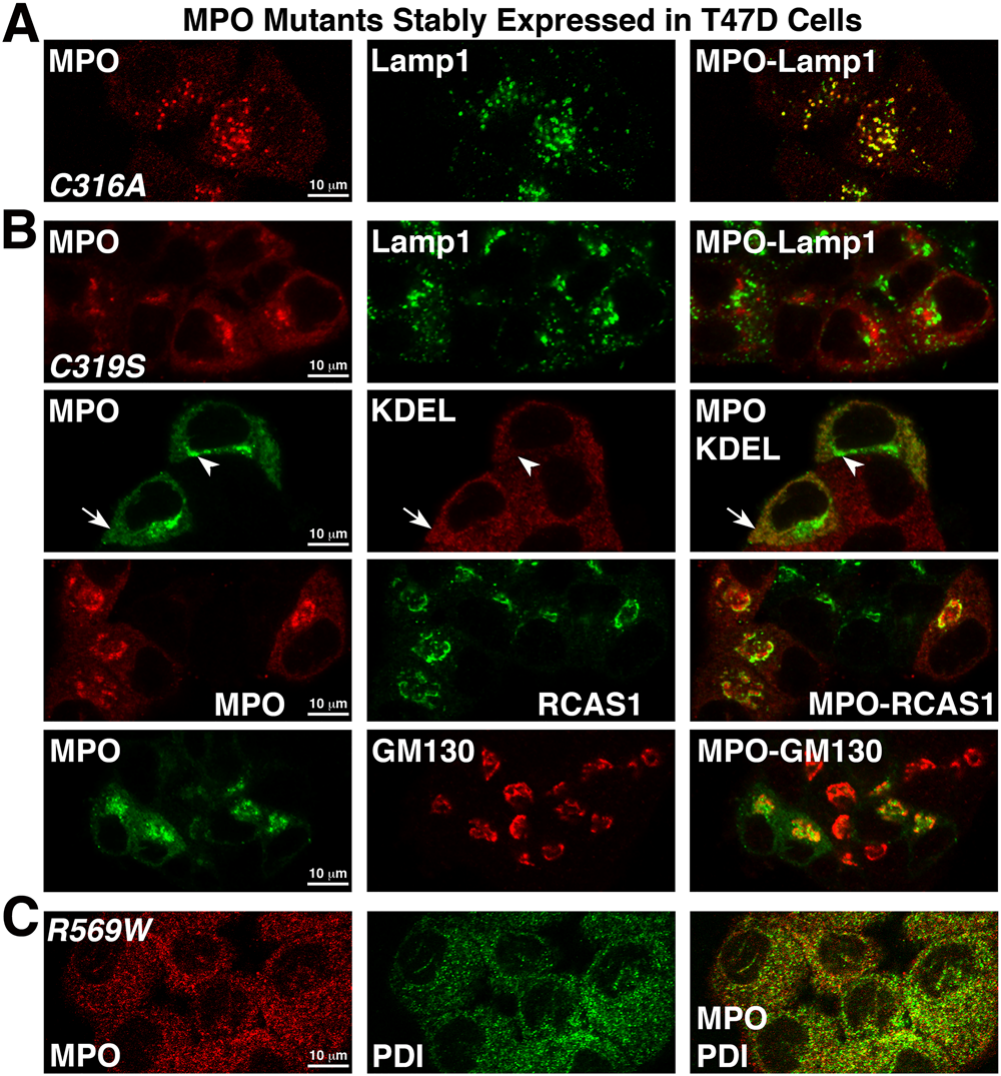
Subcellular localization of the C316A, C319S and R569W MPO mutants stably expressed in T47D cells. Cells grown on glass coverslips were fixed, permeabilized and double-labeled with polyclonal MPO antibodies and the indicated organelle marker antibody. Mounted coverslips were imaged with a 63X objective on a Zeiss LSM 710 confocal microscope. (A) T47D-C316A cells labeled with antibodies against MPO and Lamp1 and shows that the C316A mutant accumulates normally within lysosomes. (B) T47D-C319S cells labeled with antibodies against MPO (all panels) and Lamp1 (top panel), the ER marker KDEL (second panel), the trans-Golgi marker RCAS1 (third panel) and the cis-Golgi marker GM130 (fourth panel). Distinct localizations of the C319S mutant to both the ER (arrow) and Golgi (arrowhead) are indicated on the second panel. (C) T47D-R569W cells labeled with antibodies against MPO and the ER marker protein disulfide isomerase (PDI).

Immunolocalization of cysteine mutants in the T47D cell lines showed that the C316A mutant was localized most prominently in lysosomes and was indistinguishable from the staining pattern observed for wt MPO (Fig 8A). The staining pattern for the C319S (Fig 8B) and double cysteine mutants (S2A Fig) was markedly different from wt MPO and the C316A mutant. Both presented as a diffuse punctate pattern throughout the cytoplasm (arrow) that colocalized with the ER marker (KDEL) and as a brighter more concentrated staining pattern (arrowhead), that partially colocalized with membrane markers for the cis and trans-Golgi (GM130 and RCAS1), but, mostly appeared to be contained within the Golgi cisternae. These mutants displayed no significant colocalization with the lysosome marker (Lamp1) or early endosome marker (EEA1) consistent with the lack of processing observed on immunoblots. The prominent Golgi localization may also be consistent with our previous observation of complex olligosaccarides on a subset of the intracellular proMPO derived from these cell lines (Fig 5A). Since MPO appears to be secreted normally from the C319S and double mutant cell lines (Fig 7C), one might speculate that the fraction of MPO normally designated for transport to the lysosomes gets stuck in the Golgi because the C319S mutation renders it incapable of docking with a yet to be discovered receptor that introduces MPO into this specific endocytic pathway. This could result in prolonged exposure to glycosylating enzymes in the Golgi and explain why a greater fraction of the high-mannose glycosylation is converted to complex oligosaccharides for these mutants. Alternatively, conformational instability of the C319S mutant may explain both the altered glycosylation and impaired trafficking. In contrast to the cysteine mutants, the R569W mutant was only detected in the ER and combined with the very low level of secretion observed for this mutant suggests it is trapped there due to gross misfolding (Fig 8C). Therefore, the C319S and R569W mutations both appear to affect MPO folding or stability but to significantly different degrees. As further evidence of this we found that recognition of the R569W mutant by a monoclonal MPO antibody that binds a conformation-dependent epitope was severely impaired whereas binding to C319S was not affected (S2B Fig).

Because of the surprising severity and complexity of the C319S mutation we are not able to definitively say whether *inter-*molecular disulfide bond formation is necessary for the proteolytic processing and lysosomal transport of MPO. Indeed, due to their high level of sequence identity one might have expected the C319S MPO mutant to resemble the processed hemi-dimer of eosinophil peroxidase (EPO) with no functional consequence other than the lack of the *inter-*molecular disulfide bond. However, increased complex oligosaccharide content, impaired trafficking, and low specific activity of the C319S mutant coupled to the absolute family conservation at C^319^, suggest that this position is structurally or otherwise mechanistically important for events that occur prior to diversion of MPO from the Golgi towards the lysosome. The crystal structure of the mature MPO heterotetramer clearly shows the C^319^ side chain is outward facing enabling it to participate in the *inter-*molecular disulfide bond [5, 6]. If this orientation were also present in proMPO it is difficult to fathom how a change in the amino acid side from SH to OH would destabilize the structure. One possibility is that the thiol side chain of C^319^ is part of an interface that interacts with the pro-domain and stabilizes the proMPO structure for events such as glycosylation that occur early in processing. Indeed, the crystal structure of (LPO) shows that the corresponding cysteine of LPO is involved in an *intra-*molecular disulfide bond with a cysteine that is conserved within the pro-domains of MPO, EPO, LPO and thyroid peroxidase (Fig 7E) which suggests that the pro-domain may be in close proximity to C^319^ in proMPO [45]. In fact, it is possible that the analogous C^158^-C^319^ *intra-*molecular disulfide may be present in proMPO, similar to LPO; however, in the case of MPO this bond would be rearranged after exiting the ER to form the C^319^-C^319^ *inter-*molecular disulfide bond. Why mechanistically this would occur in MPO but not LPO is unclear, however, the different cleavage sites for pro-domain removal in the two proteins, occurring just C-terminal to C^159^ for MPO (and EPO) and just N-terminal to the homologous cysteine for LPO, means that inclusion of this additional *intra-*molecular disulfide bond is incompatible with removal of the MPO pro-domain at its known cleavage site. Therefore, the additional C^158^-C^319^ *intra-*molecular disulfide bond either never forms in MPO as it does in LPO or it must undergo rearrangement in a post-ER compartment. Precedent exists for such disulfide rearrangements. Post-ER disulfide rearrangement from *intra-*molecular to *inter-*molecular has been demonstrated for Von Willebrand factor oligomerization. An *intra-*molecular disulfide bond formed between the Von Willebrand factor pro-domain and a domain of the mature protein is formed in the ER, but, after exiting the ER, the chaperone activity of the pro-domain is required for rearrangement of this bond prior to or concomitant with its removal by furin protease cleavage [46, 47]. A possible model for pro-domain processing coupled to an *intra* to *inter*-molecular disulfide bond exchange of MPO is diagramed in Fig 7F and is contrasted with the lack of such an exchange for LPO.

### Testing the role of known trafficking receptors in the post-Golgi trafficking of MPO using shRNA knockdown in T47D-MPO cells

**Fig 9:**
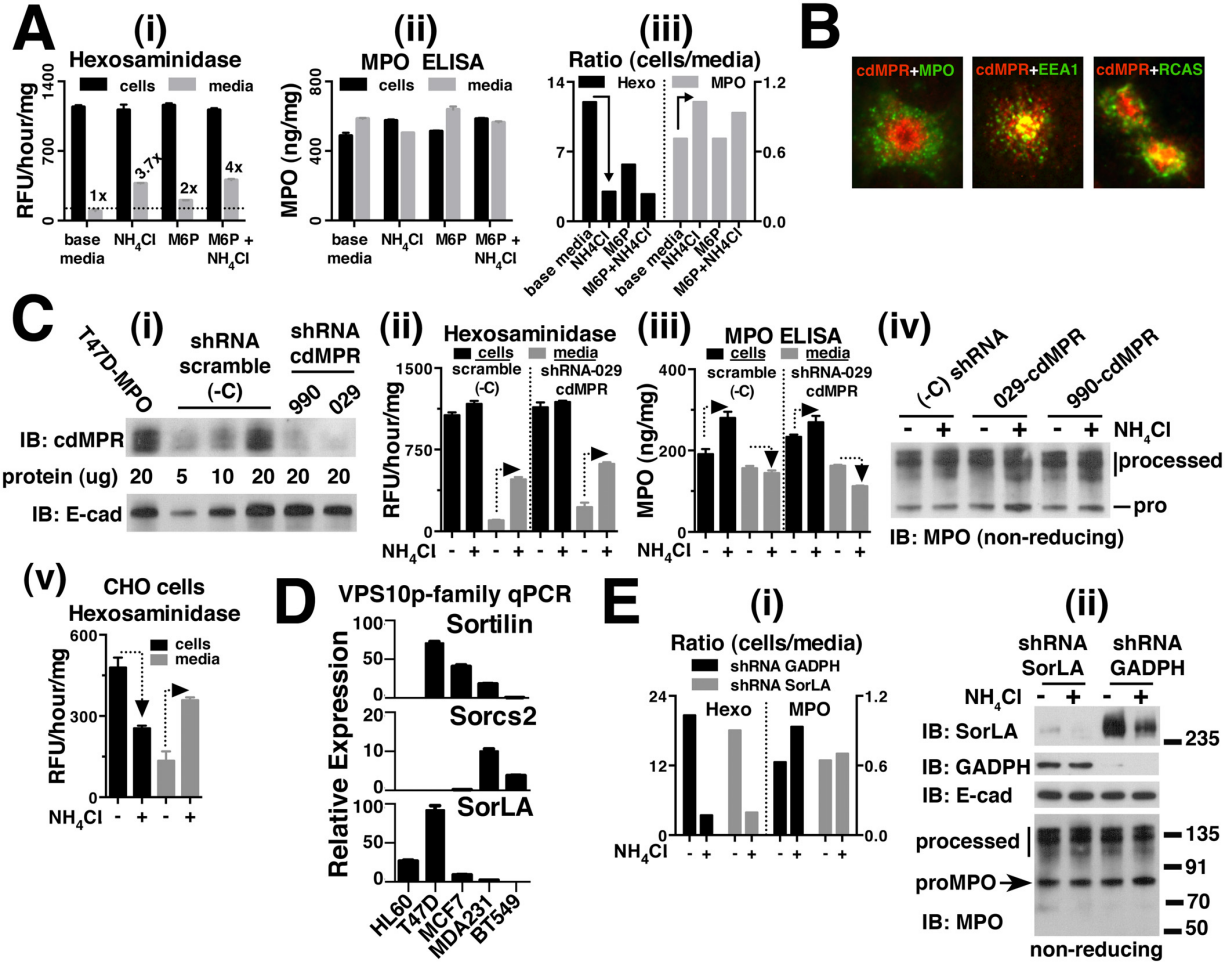
Candidate receptors queried for a role in MPO-trafficking using shRNA knockdown and NH_4_Cl in T47D-MPO cells. (A) T47D-MPO stable cells were grown for 48 hrs in media supplemented with either 10 mM NH4Cl, or 8 mM mannose + 8 mM mannose-6-phosphate (M6P) or both. The conditioned media and cell extracts were then assayed for: (i) hexosaminidase activity using a fluorogenic substrate; (ii) MPO content by ELISA to assess the treatments effects on the secretion; (iii) hexosaminidase activity and MPO concentration determined from the corresponding assays in i and ii were graphed as a ratio of that present in cell extracts divided by that present in the conditioned media (cells/media). Assay points are quadruplicate (i) and triplicate (II) measurements and plotted as mean ± SE. The data shown is representative of at least three independent experiments. (B) Immunolocalization of cation-dependent mannose-6-phosphate receptor (cdMPR) (red) with MPO, EEA1 and RCAS1 (green) in T47D-MPO cells. (C) shRNA knockdown of the cdMPR in the T47D-MPO stable cell line was carried out using lentiviral transduction as described in the “Materials and Methods”. (i) Membrane preparations derived from cells expressing either of two cdMPR shRNAs (990 & 029), a control shRNA (scramble) or the T47D-MPO parent cell line was analyzed by immunoblot with Mab-22d4 to quantify knockdown of cdMPR protein levels. Micrograms of membrane protein loaded into each lane are indicated and, as an additional loading control for membrane protein content, the blot was reprobed for E-cadheren (E-cad). (ii, iii) To examine any combined role of ciMPR and cdMPR in MPO trafficking, the cdMPR knockdown and control cell lines were grown for 44 h ± 10 mM NH_4_Cl and assayed for hexosaminidase activity and MPO content as in panel A. (iv) To examine any combined role of ciMPR and cdMPR on the processing of MPO, the cdMPR knockdown and control cell extracts from ii and iii were immunoblotted for MPO under non-reducing conditions in order to detect any changes in the relative amounts of heterotetramer and proMPO. (v) Hexosaminidase activity in CHO cells treated with and without NH_4_Cl. (D) Quantitative PCR to compare expression levels of VPS10p family members in cell lines that do (HL60, T47D and MCF7) and do not (MDA231 and BT549) efficiently form the MPO heterotetramer. Expression levels are expressed as the percent of GADPH mRNA. The experiments were repeated three times with the data from a representative experiment shown. (E) shRNA knockdown of the SorLA receptor and, as a control, GADPH was carried out using lentiviral transduction as described in the “Materials and Methods”. The selected cell lines were characterized as in panels A and C. (i) Results are summarized for each knockdown cell line as the ratio of hexosaminidase or MPO in cells/media as in panel A-iii and did not differ significantly from our observations of the parent cell line. (ii) Immunoblot analysis under non-reducing conditions of cell extracts from SorLA and GADPH shRNA-expressing cell lines to demonstrate knockdown of the respective proteins (two upper blots). Blots were reprobed with MPO antibodies to detect any changes in the relative amounts of heterotetramer and proMPO linked to decreased SorLA expression, with or without concomitant inhibition of ciMPR activity by treatment of cells with 10 mM NH_4_Cl (bottom blot). E-cadheren immunoreactivity was used as a loading control.

Many lysosomal proteins are modified with mannose-6-phosphate (M6P), which allows them to dock with M6P-receptors (MPRs) in the trans-Golgi network and traffic to the lysosome [48]. MPRs also traffic to the plasma membrane where they can pick up M6P-modified proteins secreted into the extracellular environment and traffic them to the lysosome via a more circuitous route. There are also select examples M6P-modified proteins in the extracellular environment being trafficked to lysosomes by the mannose receptor [49]. To determine whether secretion-recapture via plasma membrane-localized MPRs or the mannose receptor was a significant source of lysosomal MPO in T47D cells, we cultured the T47D-MPO cell lines for 48 hrs in the presence of a combination of free M6P and mannose. We observed no effect on the relative levels of secreted and cellular MPO. However, we did observe a two-fold increase in the amount of hexosaminidase present in the media, which suggested that a fraction of this endogenous lysosomal hydrolase travels to the lysosome via the more circuitous extracellular route in T47D cells (Fig 9A panel i).

Reagents such as NH_4_Cl that alter the pH of endosomes disrupt trafficking to the lysosome by antagonizing the binding characteristics of the cation-independent MPR (ciMPR) leading to increased extracellular secretion of cargo proteins through the constitutive secretory pathway [50]. Previous studies of MPO trafficking in HL60 cells using NH_4_Cl demonstrated that its trafficking and processing was not significantly affected by this agent, leading to the conclusion that MPO does not use MPRs to traffic to the lysosome [29, 34, 36]. We obtained similar results in our T47D-MPO cell line and observed that NH_4_Cl did not disrupt MPO trafficking to the lysosome and in fact leads to a slight decrease in the amount of secreted MPO with a concomitant increase in cellular MPO (Fig 9A panels ii & iii). In contrast to MPO, secretion of endogenous hexosaminidase was increased several fold (Fig 9A panels i & iii). These observations were qualitative validation of the similarity of MPO trafficking that occurs in recombinant T47D cells and for endogenous MPO in HL60 cells. However, neither these nor the previous observations in HL60 cells completely ruled out a role for MPRs in the trafficking and processing of MPO. A recent report suggests that, unlike the ciMPR, trafficking via the cation-dependent MPR (cdMPR) is not antagonized by NH_4_Cl [51]. Given that many lysosomal proteins can use either of the ciMPR or cdMPR for trafficking [52], we wanted to determine whether the early conclusions were valid or whether the cdMPR was in fact compensating for the ciMPR in those early studies. We immunolocalized cdMPR in our T47D-MPO expressing cells and found that, consistent with previous reports in other cell types, it partially colocalized with both the early endosomal marker EEA1 and the trans-Golgi marker RCAS1 but not significantly with MPO in the lysosomes and consistent with a role in endocytic trafficking (Fig 9B). To examine any potential compensatory role between ciMPR & cdMPR in MPO trafficking we carried out lentiviral-mediated shRNA knockdown of the cdMPR in our T47D-MPO cell line and combined the knockdown with NH_4_Cl treatment to disrupt ciMPR function. Immunoblot comparison of cdMPR protein levels from two distinct shRNA knockdown cell lines shows similar levels of knockdown (60–75%) when compared to a negative control shRNA cell line or the parent T47D-MPO cell line (Fig 9C panel i). No additional effect was observed on the relative levels of secreted and cellular MPO by knockdown of cdMPR in the presence of NH_4_Cl (Fig 9C panel iii). A modest increase was observed for basal secretion of hexosaminidase in cdMPR knockdown cells with a substantially larger increase still being observed when these cells were cultured with NH_4_Cl (Fig 9C panel ii). Immunoblot analysis of MPO species present in cell extracts from cdMPR-knockdown and control cell extracts shows that neither the knockdown nor combined treatment with NH_4_Cl negatively impacts the efficiency of MPO processing (Fig 9C panel iv). Taken together these results confirm early assertions that trafficking of MPO to lysosomes and the concomitant processing events do not require either of the two MPRs. Interestingly, we observed the expected increase in secretion of hexosaminidase when MPR function was perturbed but not a significant depletion of its intracellular levels (Fig 9C panel ii). Similar experiments in CHO cells displayed both increased hexosaminidase secretion and a concomitant decrease in its cellular levels (Fig 9C panel v). This may suggest that hexosaminidase present in T47D cells may utilize both MPR-dependent and independent mechanisms of trafficking to the lysosome. Endogenous beta-glucuronidase behaved identically to hexosaminidase in these assays (not shown).

To fully understand the molecular details of MPO trafficking and processing, the receptor(s) that chaperone it from the Golgi to the lysosome must be identified. Other than MPRs, members of the VPS10p family that in humans includes sortilin, SorLA, Sorcs 1, Sorcs 2 and Sorcs 3 have been shown to participate in the trafficking of lysosomal proteins and/or endocytic-dependent processing events [53–55]. Therefore, we carried out quantitative PCR of VPS10p family members in four breast cancer cell lines that do and do not process and store recombinant MPO as well as in HL60 cells to determine if expression of any of these family members correlated with the ability to process MPO (Fig 9D). Surprisingly, the family member most identified for its role in lysosomal trafficking, sortilin, was not expressed significantly in HL60 cells. Sorcs 1 and 3 were not detected in any of the cell lines (not shown) and Sorcs 2 expression was only significant in two of the non-processing cell lines. SorLA was the only VPS10p family member that was commonly expressed in all three cell lines that can efficiently process and store MPO; T47D, MCF7 and HL60 cells. Several attempts to create stable lentiviral-mediated SorLA knockdown cell lines in from HL60 cells were unsuccessful for technical reasons unrelated to the target. Indeed, the fragility of HL60 cells further emphasizes why availability of a more technically tractable system to test hypotheses concerning MPO is important. Accordingly, we created stable SorLA shRNA knockdown cell lines derived from the T47D-MPO stable cell line. We did not, however, observe any significant impact of SorLA knockdown on the ratio of secreted and cellular MPO when compared to a control knockdown cell line (Fig 8E panel i). We carried out these experiments on cells cultured in the absence and presence of NH_4_Cl to address the possibility that MPO trafficking is promiscuous to either MPRs or VPS10p family members even though our previous results suggested this was unlikely. SorLA knockdown did partially abrogate the modest effect of NH_4_Cl on the relative levels of MPO present in cells and media; however, secretion of MPO was not increased beyond the levels of untreated control cells (Fig 9E panel i). More importantly, no effect was observed on the levels of processed heterotetramer in SorLA knockdown cells relative to the GADPH knockdown control when MPO species were analyzed by immunoblot (Fig 9E panel ii). Interestingly, we observed that NH_4_Cl treatment by itself leads to decreased SorLA protein levels by an unknown mechanism. Taken together, these results suggest that the endocytic receptor(s) for MPO are not MPRs or a VPS10p family member. That none of these commonly used sorting receptors would be responsible for MPO trafficking is not unprecedented and non-canonical receptors have been identified for select protein cargo. The tetraspanin CD63 serves as the trafficking receptor for neutrophil elastase to azurophil granules though it has been ruled out as a candidate for MPO [56]. Carboxypeptidase E serves as the sorting receptor for several pro-hormones to the regulated secretory granules of neuroendocrine cells [13, 57]. We now have a simple expression system in which to test candidates for the role of MPO-sorting receptor if such can be identified. To that end we are comparing genome-wide expression profiles for processing and non-processing cell lines obtained from the publicly available NCI-60 database in hopes of identifying potential candidates that are common to T47D, MCF7 and HL60 cells. Indeed, the presence of the molecular machinery for MPO processing and transport in cells of non-myeloid lineage suggest that an additional trafficking paradigm, similar to MPRs and VPS10p family members, may be revealed by these efforts.

## Discussion

We describe here the in depth characterization of T47D breast cancer cells as a superior recombinant expression system for the study of the biosynthetic processing, endocytic trafficking and lysosomal storage of myeloperoxidase. Out of ten cell lines screened (not including several previously published by others), only T47D and MCF7 cells processed significant quantities of proMPO into the mature MPO heterotetramer and stored it in lysosomes in a manner similar to that of MPO expressed endogenously HL60 cells. The relatedness between T47D and MCF7 cells is noteworthy. Both cell lines are derived from estrogen receptor positive breast cancers and in mRNA expression profiles of 52 breast cancer cell lines are more closely related to each other than to any other breast cancer cell line [58]. Therefore, while it may be surprising that any non-myeloid progenitor cell line possesses the protein machinery required for MPO processing, it is not surprising that these two cell lines share this exceptional trait. We chose to characterize the recombinant T47D-MPO cell model in greater detail because of a slightly better processing capability and better expression stability of the recombinant protein over multiple cell passages. A major technical advantage of the T47D recombinant cell system over HL60 cells and previously described recombinant expression systems is the ability to study MPO trafficking in detail using fluorescent microscopy which allows cell biological and biochemical observations of the effects of mutations on the MPO biosynthetic process to be made within the same cell system. A second, incidental, but no less important advantage, is the robustness we observed of T47D cells to various cytotoxic agents. Finally, the ability to more easily employ modern molecular biology techniques, such as shRNA knockdown, to probe the molecular players that participate in MPO processing and transport is important. We took advantage of these attributes in several “proof of concept studies” to demonstrate the usefulness of the expression system and through these efforts generated new insights about post-ER processing events that result in formation and lysosomal storage of the MPO heterotetramer.

Taking advantage of the wider treatment window available for cytotoxic cathepsin inhibitors with T47D cells we were able to demonstrate that blocking the proteolytic separation of the MPO heavy and light chains does not interfere with formation of the single *inter-*molecular disulfide bond nor do these inhibitors significantly impact transport of the resulting disulfide-linked MPO dimer to the lysosome. This suggested that either the *inter-*molecular disulfide bond forms before separation of the heavy and light chains or that these events are mechanistically uncoupled. To better differentiate these possibilities we wanted to know whether or not blocking formation of the *inter-*molecular disulfide bond by mutagenesis of Cys^319^ would, in turn, have any effect on the proteolytic separation of the heavy and light chain. The answer we obtained from our cysteine mutagenesis study did not, however, further clarify the relationship between these events. In fact, out results led to additional questions regarding the importance of Cys^319^ to steps that precede its participation in the *inter-*molecular disulfide bond and suggested that Cys^319^ is important for locking proMPO into a conformation that allows both proper glycosylation and the protein-protein interactions necessary for entering the endocytic pathway that promotes these later processing events. Our data, in context with crystal structure data of LPO and MPO, led us to formulate a hypothesis that the pro-domain of MPO folds over to interact with the exposed surface of MPO containing Cys^319^, forming an C^158^-C^319^ *intra-*molecular disulfide bond. Only later, after MPO has exited the ER and during pro-domain removal is the C^158^-C^319^ *intra-*molecular disulfide bond isomerized to the *inter-*molecular C^319^-C^319^ bond that links together the two heavy chains in the heterotetramer (Fig 7F). The most direct test of our hypothesis would be a crystal structure of proMPO, though whether the MPO precursor is amenable to crystallization is an open question as, otherwise, one might have expected this to already have been reported. Additional mutagenesis complemented with a protein chemistry analysis of proMPO (free cysteine labeling coupled with mass spectrometry of proMPO digests) may also be used to substantiate this hypothesis in a less direct manner. However, whether efficient labeling to track free thiols could be achieved in an enzyme that produces oxidizing products that are themselves capable of modifying free thiols is unclear and such efforts may encounter technical hurdles.

Having multiple cell lines that process and store MPO, it will be now be possible to apply modern genome-wide expression analysis techniques to identify commonly expressed gene candidates that represent the exclusive protein machinery that allows MPO processing, using expression levels in poorly-processing cell lines as a baseline. We show proof of concept for a more focused version of this approach through identification and testing of the VPS10p family member, SorLA, as a candidate receptor for the Golgi to lysosome trafficking of MPO. Despite the correlation in expression, our data suggest that SorLA was not the endocytic trafficking receptor for MPO, though it is possible that sortilin, which is not expressed in HL60 cells but is expressed highly in T47D cells, compensated for the loss of SorLA in our shRNA knockdown experiments. Nonetheless, we have established a useful paradigm for future identification of such a receptor. That such a receptor protein exists and that its expression is tightly regulated in a tissue-specific manner is strongly supported by the lack of significant processing or storage of MPO in all but promyelocytes and estrogen receptor positive breast cancer cells. It is also of interest that T47D and MCF7 cells retain many of the epithelial characteristics of normal breast epithelia that are lost in the more mesenchymal orientated, estrogen receptor-negative cell lines such as MDA231, MDA468 and BT549 [58]. This leaves open the possibility that the proposed endocytic receptor for MPO has not evolved for the singular purpose of MPO processing, but may function in other cell types specialized to produce large quantities of select proteins for release into the extracellular environment, such as milk proteins produced by breast epithelial cells during lactation. Identification of the specific proteins responsible for each facet of MPO processing and trafficking in the T47D cells system is tractable and will facilitate our understanding of how these events occur in vivo. However, as is the case when using any cell model based on recombinant protein expression, confirmation of any protease, endosome receptor or other protein’s role in producing mature MPO will eventually have to be confirmed through targeted gene disruption in animal models, HL60 cells or both.

## Supporting Information

S1 Fig Subcellular localization of MPO stably expressed in several cell types. Cells grown on coverslips were fixed, permeabilized, double-labeled with the indicated antibodies and imaged with the 100x oil objective on a conventional fluorescent microscope. (A) Fluorescent images of the T47D-MPO stable cell line labeled with antibodies against MPO (green) and the ER marker KDEL (red). Colocalization is indicated by the arrowhead. (B) Fluorescent images of the MCF7-MPO cell line labeled with antibodies against MPO and Lamp1 (upper panel), the early endosome marker EEA1 (middle panel) and ER marker KDEL (bottom panel) with colors as indicated in each panel. (C) Fluorescent images of the MDA468-MPO cell line labeled with antibodies against MPO (red) and Lamp1 (green). (D) Neuro2A-MPO cell line labeled with antibodies against MPO (red) and Lamp1 (green). MPO staining concentrated in the termini of cell processes is indicated by arrowheads. The termini of Neuro2A cells contain regulated secretory granules that are distinct in origin from lysosome granules. Blue color represents DAPI staining of nuclei in all color images.
(TIFF)

S2 Fig Confocal images of the T47D C316A-C319S MPO double mutant with additional subcellular markers. Selective binding to a monoclonal antibody provides evidence that folding of the R569W mutant is severely compromised in comparison to the cysteine mutants of MPO. (A) Cells grown on coverslips were double-labeled with the indicated antibodies and imaged with a 63x oil objective on a Zeiss LSM 710 confocal microscope. (A) Fluorescent images of the T47D C316A-C319S double mutant cell line labeled with goat polyclonal antibodies against MPO (red) and a rabbit antibody against the early endosome marker EEA1 (green) (upper panel) or Mab-16E3 against MPO (red) and rabbit antibody against the trans-Golgi marker RCAS1 (green) (lower panel). (B) Cell extracts derived from T47D stable cell lines expressing wt or mutant MPO were incubated on duplicate ELISA plates coated with multi-epitope rabbit polyclonal anti-MPO antibody. Bound MPO was detected either with HRP-conjugated Mab-16E3 or with an HRP-conjugated multi-epitope goat polyclonal antibody. Both Mab-16E3 and the goat polyclonal detection antibodies yield identical measurements of MPO concentration for wt MPO and the cysteine mutants, whereas binding of Mab-16E3 to the R569W mutant is significantly impaired relative to the goat polyclonal. Assay points were in triplicate and plotted as the mean ± SE. Results are representative of two independent experiments. Data for each cell line was normalized to the highest value before plotting to compensate for different expression levels between cell lines.
(TIFF)
