# Supplementary figures and images for: T47D Cells Expressing Myeloperoxidase Are Able to Process, Traffic and Store the Mature Protein in Lysosomes: Studies in T47D Cells Reveal a Role for Cys319 in MPO Biosynthesis that Precedes Its Known Role in Inter-Molecular Disulfide Bond Formation
Source: PLoS One. 2016 Feb 18;11(2):e0149391. doi: 10.1371/journal.pone.0149391 (PMC4758715; doi:10.1371/journal.pone.0149391)

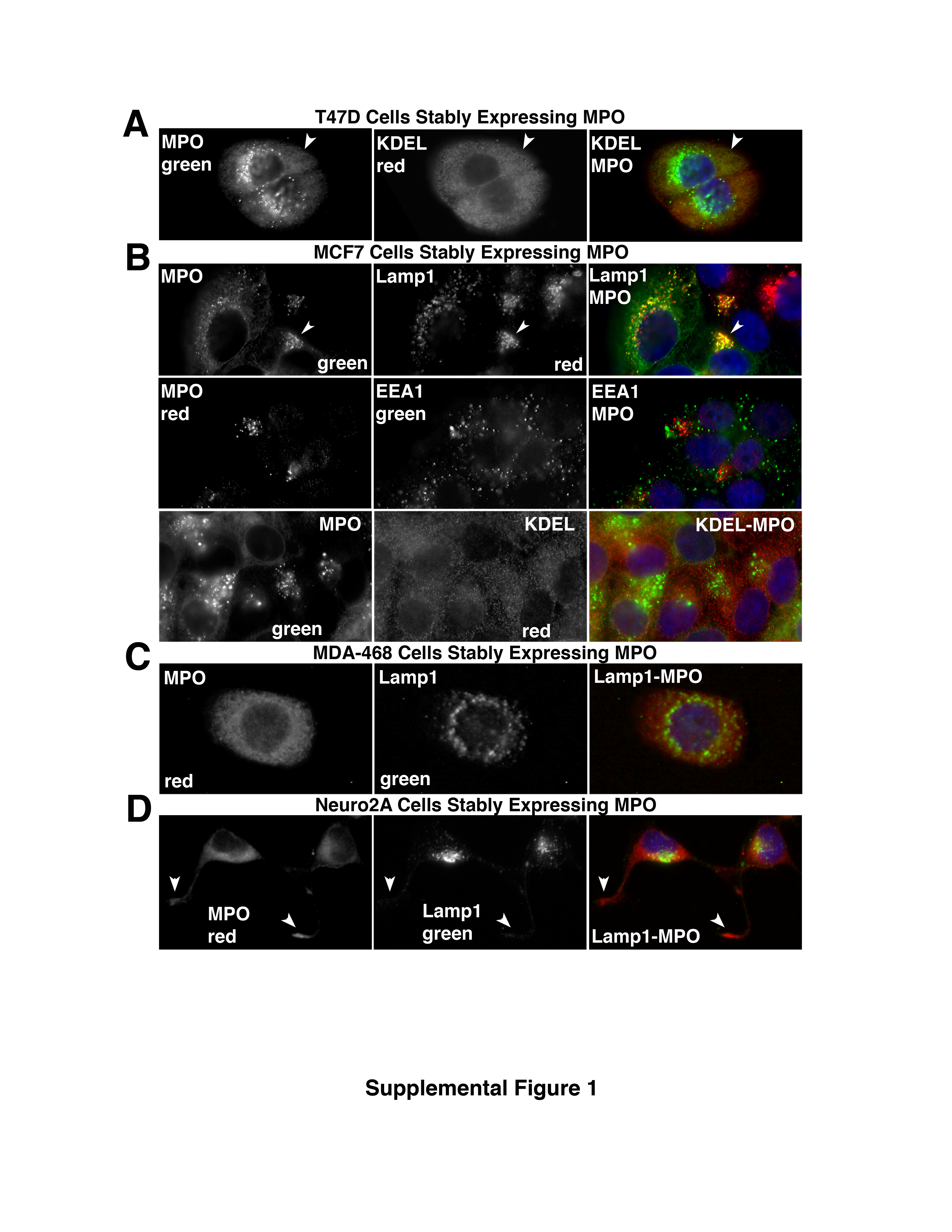

Supplement: S1 Fig — Cells grown on coverslips were fixed, permeabilized, double-labeled with the indicated antibodies and imaged with the 100x oil objective on a conventional fluorescent microscope. (A) Fluorescent images of the T47D-MPO stable cell line labeled with antibodies against MPO (green) and the ER marker KDEL (red). Colocalization is indicated by the arrowhead. (B) Fluorescent images of the MCF7-MPO cell line labeled with antibodies against MPO and Lamp1 (upper panel), the early endosome marker EEA1 (middle panel) and ER marker KDEL (bottom panel) with colors as indicated in each panel. (C) Fluorescent images of the MDA468-MPO cell line labeled with antibodies against MPO (red) and Lamp1 (green). (D) Neuro2A-MPO cell line labeled with antibodies against MPO (red) and Lamp1 (green). MPO staining concentrated in the termini of cell processes is indicated by arrowheads. The termini of Neuro2A cells contain regulated secretory granules that are distinct in origin from lysosome granules. Blue color represents DAPI staining of nuclei in all color images. (TIFF) [file pone.0149391.s001.tiff]

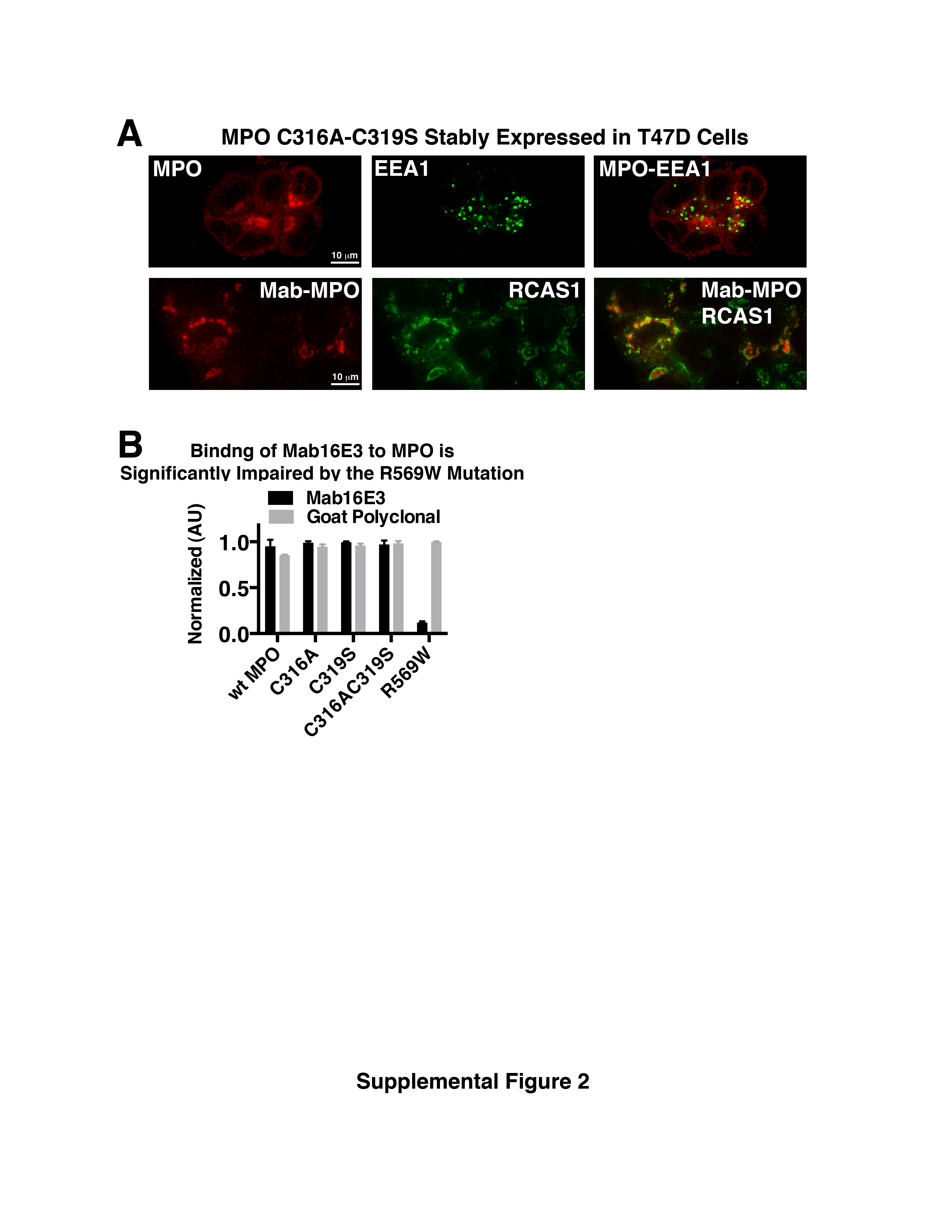

Supplement: S2 Fig — (A) Cells grown on coverslips were double-labeled with the indicated antibodies and imaged with a 63x oil objective on a Zeiss LSM 710 confocal microscope. (A) Fluorescent images of the T47D C316A-C319S double mutant cell line labeled with goat polyclonal antibodies against MPO (red) and a rabbit antibody against the early endosome marker EEA1 (green) (upper panel) or Mab-16E3 against MPO (red) and rabbit antibody against the trans-Golgi marker RCAS1 (green) (lower panel). (B) Cell extracts derived from T47D stable cell lines expressing wt or mutant MPO were incubated on duplicate ELISA plates coated with multi-epitope rabbit polyclonal anti-MPO antibody. Bound MPO was detected either with HRP-conjugated Mab-16E3 or with an HRP-conjugated multi-epitope goat polyclonal antibody. Both Mab-16E3 and the goat polyclonal detection antibodies yield identical measurements of MPO concentration for wt MPO and the cysteine mutants, whereas binding of Mab-16E3 to the R569W mutant is significantly impaired relative to the goat polyclonal. Assay points were in triplicate and plotted as the mean ± SE. Results are representative of two independent experiments. Data for each cell line was normalized to the highest value before plotting to compensate for different expression levels between cell lines. (TIFF) [file pone.0149391.s002.tiff]
